# Mechanism-Based Pharmacokinetic/Pharmacodynamic Modeling for Iron-Regulated Hematopoietic Stem and Progenitor Cells’ Commitment toward Erythroid and Megakaryocytic Lineages

**DOI:** 10.1021/acsptsci.5c00097

**Published:** 2025-05-30

**Authors:** Kangna Cao, Xiaoqing Fan, Raymond S. M. Wong, Xiaoyu Yan

**Affiliations:** 1 Guangdong-Hong Kong-Macao Joint Laboratory for New Drug Screening, School of Pharmacy, 26451 The Chinese University of Hong Kong, Hong Kong SAR, P. R. China; 2 Division of Hematology, Department of Medicine and Therapeutics, Faculty of Medicine, 26451 The Chinese University of Hong Kong, Hong Kong SAR, P. R. China

**Keywords:** Iron, HSPCs, erythropoiesis, thrombopoiesis, anemia, mechanism-based PK/PD model

## Abstract

Iron replenishment is a cornerstone therapy for anemia in diverse diseases. While its role in erythrocyte hemoglobinization is well-established, the broader impact of iron on other aspects of hematopoiesis, such as thrombopoiesis, remains poorly understood. In this study, we demonstrate that iron plays a regulatory role in the commitment of hematopoietic stem and progenitor cells (HSPCs) toward erythroid and megakaryocytic lineages. Using colony-forming unit assays and flow cytometry, we observed that iron increases the proportion of erythroid cells while reducing the proportion of megakaryocytic cells. Transcriptomic profiling and functional output analyses identified the MAPK/ERK pathway as a critical mediator of iron-regulated HSPCs’ commitment. Corroborating *in vitro* findings, rats with iron deficiency anemia exhibited continuously elevated platelets and decreased red blood cell counts, while intravenous iron supplementation reversed these effects. This effect of iron was enhanced in combination with erythropoietin, a key cytokine in erythropoiesis. A mechanism-based pharmacokinetic/pharmacodynamic model was developed to quantify the impact of iron on the two lineages. The dynamic interplay between iron levels and the development of erythropoiesis and thrombopoiesis was accurately recapitulated in rats. The model was further extrapolated to humans and validated with clinical data. Overall, this work not only provides functional insights into the pivotal role of iron in erythropoiesis and thrombopoiesis but also holds translational implications for optimizing iron therapy in anemia and potentially other hematologic conditions where erythropoiesis and thrombopoiesis are affected.

## Graphical abstract

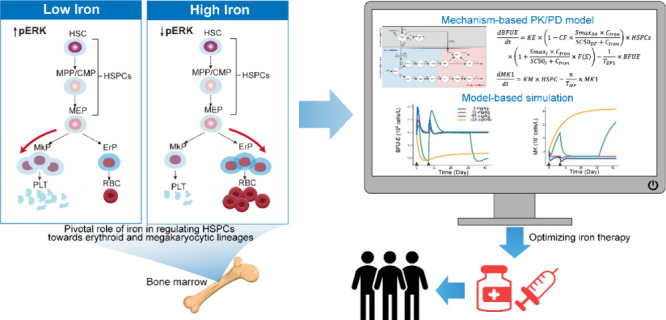

As an essential constituent of hemoglobin (HGB), iron is vital for supporting erythropoiesis. Iron deficiency can result in anemia, which is highly prevalent worldwide.^1^ Intravenous iron supplementation, either alone or in combination with erythropoiesis-stimulating agents (ESAs), has become a standard treatment for anemia in various conditions such as chronic kidney disease, inflammatory bowel disease, and heart failure.

While the importance of iron for red blood cell (RBC) formation is widely acknowledged, its impact on other aspects of hematopoiesis such as thrombopoiesis warrants exploration. Platelets (PLTs), or thrombocytes, serve as key mediators of hemostasis and contribute to diverse physiological processes, including inflammation, wound healing, and immune responses. Clinical reports have linked iron deficiency to elevated platelet counts, yet the underlying mechanism remains poorly understood. Interestingly, thrombocytosis induced by iron deficiency appears to be independent of thrombopoietin (TPO), a hormone that regulates platelet production, as well as other common thrombopoietic cytokines such as IL-11 and IL-6.^2^ Additionally, while erythropoietin (EPO) has been proposed as a potential mediator of increased platelet counts in iron deficiency anemia (IDA), our previous research suggests that EPO, under conditions of iron repletion, can inhibit platelet production in a dose-dependent manner.^3^ Thus, it is plausible that iron deficiency itself may drive the elevation of platelets in IDA patients rather than through stimulation by cytokines. Notably, platelets are derived from megakaryocytes, which share the common bipotent progenitor cell, megakaryocytic-erythroid progenitors (MEPs), with erythrocytes.^4^ Based on these lines of information, it is speculated that, apart from its role in supporting HGB production in RBC, iron may influence erythropoiesis and thrombopoiesis by regulating HSPCs’ commitment toward the two lineages. If so, platelet count could emerge as a crucial biomarker necessitating close monitoring in anemia management with iron therapy, especially given the established connections between thrombocytosis and thrombotic risk in iron deficiency.^5,6^

In this study, we clarified the pivotal role of iron in directing HSPCs toward erythroid and megakaryocytic lineages, alongside its interplay with EPO, through both *in vitro* and *in vivo* studies in rats. Importantly, we developed a mechanism-based pharmacokinetic/pharmacodynamic (PK/PD) model that quantifies iron’s impact on these two lineages and characterizes the dynamic processes of intercompartment progenitor cells and lineage outputs under varying iron levels through model-based simulation. This model was further validated in humans, offering potential clinical utility for optimizing iron therapy in anemia and possibly other hematologic conditions where erythropoiesis and thrombopoiesis are affected.

## Methods

### Animals

The animal experiments received approval from the Animal Experimentation Ethics Committee of the Chinese University of Hong Kong (22-241-GRF). The IDA model in rats was employed as previously described.^7,8^ Further details are provided in the Supporting Information.

### Proliferation and Differentiation of Rat HSPCs

This study employed enriched HSPCs from rats to create an *in vitro* system for examining the effects of varying iron concentrations, alone and in combination with recombinant human erythropoietin (rHuEPO), on the proliferation and differentiation of HSPCs.^3^ The cells were cultured in StemSpan SFEM (STEMCELL, Canada) at 37 °C with 5% CO_2_. As the medium contained holo-transferrin (holo-trf), different iron concentrations were achieved by adding varying amounts of additional holo-trf or iron chelator deferiprone (DFP). Further details are provided in the Supporting Information.

### Colony-Forming Unit (CFU) Assay

CFU assays were conducted to investigate the effect of iron levels, either alone or in combination with rHuEPO, on the differentiation of HSPCs. A semisolid medium (MethoCult SF M3436, STEMCELL) was used for the growth of colony-forming unit-erythroid (CFU-E) and burst-forming unit-erythroid (BFU-E) as per the manufacturer’s guidelines. For colony-forming unit-megakaryocyte (CFU-MK) assays, progenitors were cultured following a method previously reported.^3^

### Flow Cytometric Analysis

The differentiation of HSPCs into megakaryocytic and erythroid lineages was also assessed using the combination of two biomarkers: CD71 and CD41.^9–11^ Details are provided in the Supporting Information.

### PK/PD Studies of Intravenous Iron in IDA Rats

Ferric carboxymaltose (Ferrinject, FCM; Vifor Pharma, Glattbrugg, Switzerland) was chosen as an intravenous iron source due to its proven efficacy and safety in rapidly replenishing iron stores as well as its ability to correct IDA in a murine model. Oral iron preparation was not included to exclude the influence of absorption. Rats with IDA were randomized to three different doses of FCM alone (3, 15, or 90 mg/kg, once weekly [QW]) or in combination with rHuEPO (rHuEPO 450 IU/kg, thrice weekly [TIW] + FCM 3/15/90 mg/kg, QW) or rHuEPO alone (450 IU/kg, TIW) (*n* = 3 per group). Rats with IDA received FCM or rHuEPO for 2 weeks intravenously through the tail vein. The FCM treatment schedule and dosage have been based on data from previous literature, which suggest that an intermediate dose of 15 mg/kg once weekly is sufficient to correct iron deficiency.^8,12,13^ A higher dose and a lower dose of FCM were selected based on the consideration that PD is usually nonlinear and its thorough evaluation requires multiple dose levels. The rHuEPO regimen of 450 IU/kg IV thrice weekly was selected based on the clinically relevant dose and our previous publications.^3^ Two PK samples (5 min and 48 h after administration) were collected after each dose to minimize the effect of blood loss on hematological parameters. Hematological parameters were monitored through blood sampling from the tail vein on days 0, 2, and 4 every week until 7 weeks after the first dose.

### RNA-Sequencing and Statistical Analysis of RNA-seq Data

Library construction and sequencing were carried out by LC-BIO Biotech Ltd. (Hangzhou, China). Differential expression analysis between each pair of groups was performed using DESeq2 (version 1.22.2). Genes with a false discovery rate (FDR) below 0.05 and an absolute fold change ≥ 1.5 were considered differentially expressed genes (DEGs). To obtain detailed information on the effect of iron on the transcriptome of HSPCs, DEGs identified between the three groups were clustered into eight profiles based on gene expression patterns using the Short Time-series Expression Miner software (version 1.3.11).^14^ Clusters with an adjusted *P* value < 0.05 were considered significant. Only DEGs in profiles that were statistically significant in STEM analysis and exhibited consistent upregulation or downregulation were selected for subsequent Gene Ontology (GO) and Kyoto Encyclopedia of Genes and Genomes (KEGG) enrichment analysis. Data handling was performed with the R statistical software (version 4.3.0), and bioinformatic analysis was conducted using the OmicStudio tools (https://www.omicstudio.cn/tool.)

### Development of a Mechanism-Based PK/PD Model

To quantify the effect of iron on erythropoietic and thrombopoietic conditions, a mechanism-based PK/PD model was developed using NONMEM (version 7.5.0, ICON plc).

#### PK Model

The combination data of sparse sampling in this study (0, 5 min, and 48 h after each dosing) and intensive sampling (0, 1, 2, 4, 6, 8, 12, 24, and 48 h after single dosing) in a previous publication^15^ were used for PK model development. The pharmacokinetic profile of serum iron was fitted by a two-compartment model with linear elimination. The differential equations were as follows:

$$\frac{dA_{1}(t)}{dt}={\mathrm{KIN}}_{\mathrm{Iron}}-k_{\mathrm{CP}}\cdot A_{1}(t)+k_{\mathrm{PC}}\cdot A_{2}(t)-k_{\mathrm{el}}\cdot A_{1}(t)$$ 1

$$\frac{dA_{2}(t)}{dt}=k_{\mathrm{CP}}\cdot A_{1}(t)-k_{\mathrm{PC}}\cdot A_{2}(t)$$ 2

*A*_1_(*t*) and *A*_2_(*t*) represent the amounts of iron in the central and peripheral compartments, respectively. KIN_Iron_ is the input rate of iron. *k*_el_ is the constant rate of linear elimination, and *k*_CP_ and *k*_PC_ are the intercompartmental rate constants from the central compartment to the peripheral compartment and from the peripheral compartment to the central compartment, respectively. *k*_el_, *k*_CP,_ and *k*_PC_ are parametrized according to the following equations:

$$k_{\mathrm{el}}=\frac{\mathrm{CL}}{V_{1}}$$ 3

$$\begin{array}{c} k_{\mathrm{CP}}=\frac{Q_{\mathrm{CP}}}{V_{1}} \end{array}$$ 4

$$\begin{array}{c} k_{\mathrm{PC}}=\frac{Q_{\mathrm{PC}}}{V_{2}} \end{array}$$ 5

where CL is the clearance of iron and *V*_1_ and *V*_2_ represent the volumes of the central and peripheral compartments of iron, respectively. *Q*_CP_ and *Q*_PC_ are the clearances from the central to the peripheral compartment and from the peripheral to the central compartment, respectively. Considering that there was no regulated mechanism for the excretion of iron, clearance was fixed at 0.0001.^16^ At time zero of the observation period, the system was assumed to be at its physiological steady state, resulting in the following equations:

$$\begin{array}{c} A_{1}(0)=\frac{{\mathrm{KIN}}_{\mathrm{Iron}}}{k_{\mathrm{el}}} \end{array}$$ 6

$$\begin{array}{c} A_{2}(0)=\frac{{\mathrm{KIN}}_{\mathrm{Iron}}}{k_{\mathrm{el}}}\cdot \frac{k_{\mathrm{CP}}}{k_{\mathrm{PC}}} \end{array}$$ 7

#### PD Model

Maturation-structured cytokinetic transit compartment models based on ordinary differential equations were utilized to characterize erythropoiesis^17^ and thrombopoiesis,^18^ describing the delays in cell maturation from progenitor cells to specific blood cells. Each compartment corresponds to a cell population at various stages of development.^18,19^

For erythropoiesis, the model consists of a sequence of compartments, including HSPCs, BFU-E, CFU-E, normoblasts (NORs), and reticulocytes (RETs), which ultimately develop into RBCs. The differential equation of the HSPCs compartment was described as follows:

$$\begin{array}{c} \frac{d\mathrm{HSPCs}}{dt}=\mathrm{KIN}-\mathrm{KE}\cdot \left(1-\mathrm{DF}\cdot \left(1-\frac{S\max_{\mathrm{DF}}\cdot C_{\mathrm{Iron}}}{\mathrm{SC}50_{\mathrm{DF}}+C_{\mathrm{Iron}}}\right)\right)\cdot \mathrm{HSPCs}\cdot \left(1+\frac{S\max_{\mathrm{Iron}}\cdot C_{\mathrm{Iron}}}{\mathrm{SC}50_{\mathrm{Iron}}+C_{\mathrm{Iron}}}\cdot F\left(S\right)\right)\\ -\mathrm{KM}\cdot \mathrm{HSPCs} \end{array}$$ 8

where KIN is the zero-order rate constant for the production of HSPCs. KE and KM are the first-order rate constants for HSPCs to differentiate into erythroid and MK lineages, respectively. *C*_Iron_ is the change of serum iron concentration from the baseline; DF is a disease progression factor. Smax_DF_ is the maximal correction of DF after intravenous iron supplement, and it is fixed as 1. SC50_DF_ is the *C*_Iron_ that has a half-maximum effect on the DF correction. Smax_Iron_ is the maximal stimulus of iron on promoting HSPCs toward BFU-E, and SC50_Iron_ is the *C*_Iron_ that has a half-maximum effect. According to the *in vitro* and *in vivo* results, only high iron levels promote HSPCs toward BFU-E. Therefore, iron promotes HSPCs toward erythroid lineage according to an on-and-off function, where Cutoff_Iron_ is the cutoff value of *C*_Iron_ to stimulate HSPCs:

$$\begin{array}{c} F(S)=\{\begin{array}{c} 1,\;\mathrm{if}\;C_{\mathrm{Iron}}>{\mathrm{Cutoff}}_{\mathrm{Iron}}\\ 0,\;\mathrm{if}\;C_{\mathrm{Iron}}\leq {\mathrm{Cutoff}}_{\mathrm{Iron}} \end{array} \end{array}$$ 9

The compartments of erythroid lineage were characterized as follows:

$$\frac{d\mathrm{BFUE}}{dt}=\mathrm{KE}\cdot \left(1-\mathrm{DF}\cdot \left(1-\frac{S\max_{\mathrm{DF}}\cdot C_{\mathrm{Iron}}}{\mathrm{SC}50_{\mathrm{DF}}+C_{\mathrm{Iron}}}\right)\right)\cdot \mathrm{HSPCs}\cdot \left(1+\frac{S\max_{\mathrm{Iron}}\cdot C_{\mathrm{Iron}}}{\mathrm{SC}50_{\mathrm{Iron}}+C_{\mathrm{Iron}}}\cdot F\left(S\right)\right)-\frac{1}{T_{\mathrm{EP}1}}\cdot \mathrm{BFUE}$$ 10

$$\frac{d\mathrm{CFUE}}{dt}=2^{\mathrm{MCFU}}\cdot \frac{1}{T_{\mathrm{EP}1}}\cdot \mathrm{BFUE}-\frac{1}{T_{\mathrm{EP}2}}\cdot \mathrm{CFUE}$$ 11

$$\frac{d\mathrm{NOR}}{dt}=2^{\mathrm{MNOR}}\cdot \frac{1}{T_{\mathrm{EP}2}}\cdot \mathrm{CFUE}-\frac{1}{T_{\mathrm{EP}3}}\cdot \mathrm{NOR}$$ 12

$$\frac{d\mathrm{RET}}{dt}=\frac{1}{T_{\mathrm{EP}3}}\cdot \mathrm{NOR}-\frac{1}{T_{\mathrm{RET}}}\cdot \mathrm{RET}$$ 13

$$\frac{d\mathrm{RBC}}{dt}=\frac{1}{T_{\mathrm{RET}}}\cdot \mathrm{RET}-\frac{1}{T_{\mathrm{RBC}}}\cdot \mathrm{RBC}$$ 14

where 2^MCFU^ and 2^MNOR^ are factors that indicate the number of CFU-E cells generated by a single BFU-E and the number of NORs produced by a single CFU-E cell, respectively.^19^ *T*_EP_ represents the average time required for precursors to transition to the next cell population. *T*_RET_ and *T*_RBC_ denote the mean residence time for RETs and RBCs, respectively.^17^ To simplify the model parameters, *T*_EP_ was assumed to be equal to *T*_RET_.

HGB is steadily produced at zero-order rate constant KIN_HGB_ and removed with first-order rate constant KOUT_HGB_ as follows:

$$\begin{array}{c} \frac{d\mathrm{HGB}}{dt}={\mathrm{KIN}}_{\mathrm{HGB}}\cdot \left(1+\frac{S\max_{\mathrm{HGB}}\cdot C_{\mathrm{Iron}}}{\mathrm{SC}50_{\mathrm{HGB}}+C_{\mathrm{Iron}}}\right)-{\mathrm{KOUT}}_{\mathrm{HGB}}\cdot \mathrm{HGB} \end{array}$$ 15

Smax_HGB_ denotes the maximal stimulus of iron on HGB production, and SC50_HGB_ is the *C*_Iron_ that has a half-maximum effect on HGB production. The elimination rate of HGB was assumed to match that of RBC to simplify the number of parameters. At the start of the observation period (time zero), the system was presumed to be at its physiological steady state, resulting in the following equation:

$$\begin{array}{c} {\mathrm{KIN}}_{\mathrm{HGB}}=\frac{{\mathrm{HGB}}_{0}}{T_{\mathrm{RBC}}} \end{array}$$ 16

Platelet production involves a sequence of aging compartments representing MK precursor cells in the bone marrow (MK_*n*_, where *n* = 10), with transition rates denoted by *n*/*T*_MP_. Each compartment was calculated according to the equations given below:

$$\begin{array}{c} \frac{d\mathrm{MK}1}{dt}=\mathrm{KM}\cdot \mathrm{HSPC}-\frac{n}{T_{\mathrm{MP}}}\cdot \mathrm{MK}1 \end{array}$$ 17

$$\begin{array}{c} \frac{d{\mathrm{MK}}_{i}}{dt}=\frac{n}{T_{\mathrm{MP}}}\cdot ({\mathrm{MK}}_{i-1}-{\mathrm{MK}}_{i})\;i=2,...,n \end{array}$$ 18

Likewise, PLT_*n*_ (where *n* = 10) are platelets in the blood with transition rates indicated by *n*/*T*_PLT_.

$$\begin{array}{c} \frac{d{\mathrm{PLT}}_{1}}{dt}=\mathrm{CF}\cdot \frac{n}{T_{\mathrm{MP}}}\cdot {\mathrm{MK}}_{n}-\frac{n}{T_{\mathrm{PLT}}}\cdot {\mathrm{PLT}}_{1} \end{array}$$ 19

$$\begin{array}{c} \frac{d{\mathrm{PLT}}_{i}}{dt}=\frac{n}{T_{\mathrm{PLT}}}\cdot ({\mathrm{PLT}}_{i-1}-{\mathrm{PLT}}_{i})\;i=2,...,n \end{array}$$ 20

where *T*_MP_ and *T*_PLP_ represent the mean lifespan of MK precursor cells and platelets, respectively. CF denotes the factor representing the average number of platelets produced by a single MK cell and was fixed at 4000.^18^ Platelets were modeled as the total platelet counts across all PLT compartments.

$$\begin{array}{c} \mathrm{PLT}={\mathrm{PLT}}_{1}+\cdot \cdot \cdot +{\mathrm{PLT}}_{n} \end{array}$$ 21

The baseline equations and secondary parameters, determined by the steady state, can be utilized to simplify the model parameters as follows:

$$\begin{array}{c} {\mathrm{RET}}_{0}={\mathrm{RBC}}_{0}\cdot T_{\mathrm{RET}}/(T_{\mathrm{RBC}}+T_{\mathrm{RET}}) \end{array}$$ 22

$$\begin{array}{c} {\mathrm{NOR}}_{0}={\mathrm{RET}}_{0}\cdot T_{\mathrm{EP}3}/T_{\mathrm{RET}} \end{array}$$ 23

$$\begin{array}{c} {\mathrm{CFUE}}_{0}={\mathrm{RET}}_{0}\cdot T_{\mathrm{EP}2}/(T_{\mathrm{RET}}\cdot 2^{\mathrm{MNOR}}) \end{array}$$ 24

$$\begin{array}{c} {\mathrm{BFUE}}_{0}={\mathrm{RET}}_{0}\cdot T_{\mathrm{EP}1}/(T_{\mathrm{RET}}\cdot 2^{\mathrm{MNOR}}\cdot 2^{\mathrm{MCFU}}) \end{array}$$ 25

$$\begin{array}{c} {\mathrm{HSPC}}_{0}={\mathrm{BFUE}}_{0}/(T_{\mathrm{EP}1}\cdot \mathrm{KE}) \end{array}$$ 26

$$\begin{array}{c} {\mathrm{MK}}_{0}=T_{\mathrm{MP}}\cdot {\mathrm{PLT}}_{0}/(\mathrm{CF}\cdot T_{\mathrm{PLT}}\cdot 10) \end{array}$$ 27

$$\begin{array}{c} \mathrm{KIN}={\mathrm{HSPC}}_{0}\cdot (\mathrm{KM}+\mathrm{KE}) \end{array}$$ 28

$$\begin{array}{c} \mathrm{KM}=\frac{{\mathrm{PLT}}_{0}}{\mathrm{CF}\cdot T_{\mathrm{PLT}}\cdot {\mathrm{HSPC}}_{0}} \end{array}$$ 29

The naive pooled data modeling approach was employed, treating all individual data as if they originated from a single unique individual. Residual variabilities of RBC, PLT, and HGB were evaluated separately. Various residual error models were explored, including an additive error model, a proportional error model, and a combined error model. Ultimately, the residual variability for RBC and PLT was best described by a proportional error model, while the HGB variability was best captured by an additive error model. Ordinary differential equations were solved using the ADVAN13 subroutine, and parameter estimation was performed using the expectation–maximization (EM) algorithm (stochastic approximation EM followed by important sampling EM).

### Extrapolation of the Model to Humans

Based on the original model structure in rats, the PK model was refitted with published data in IDA patients administered with FCM at a single dose of 100, 500, 800, or 1000 mg or placebo.^20^ The ordinary differential equations were solved by the ADVAN13 subroutine, and the first-order conditional estimation method with the interaction (FOCEI) algorithm was used for parameter estimation. The PD model was adapted for humans by translating key lineage-specific hematological parameters, including *T*_RET_, *T*_RBC_, RBC_0_, *T*_MP_, *T*_PLT_, PLT_0_, and HGB_0_, from rats to humans.^21–23^ The specific values and their corresponding references are listed in Table S1. The adapted model was subsequently used to simulate the dynamics of HGB and PLT counts under various intravenous iron treatments in IDA patients and validated with clinically observed data.^24,25^

### Statistical Analysis

Statistical analyses were conducted using GraphPad Prism (version 6.0.2), with a *P* value < 0.05 considered statistically significant for analysis of variance or Student’s two-tailed *t* test. Data are expressed as the mean plus standard deviation (S.D.) unless otherwise specified.

## Results

### A High Concentration of Iron Drives HSPCs into the Erythroid Lineage, Whereas a Low Concentration of Iron Promotes Megakaryopoiesis

We first assessed the impact of iron on the expansion and differentiation of HSPCs in an *in vitro* system. As shown in Figure 1A, there was a bell-shaped relationship between the iron availability and HSPC expansion. This observation was further supported by the U-shaped relationship observed between the iron concentration and apoptosis levels (Figure 1B). Additionally, a U-shaped relationship was observed between the iron concentration and reactive oxygen species (ROS) levels (Figure 1C). Specifically, ROS levels remained stable across the five middle iron concentration groups. However, consistent with previous studies,^26^ higher iron concentrations resulted in increased ROS levels and reduced cell yields, while excessively low iron concentrations induced survival stress.^27^

**Figure 1:**
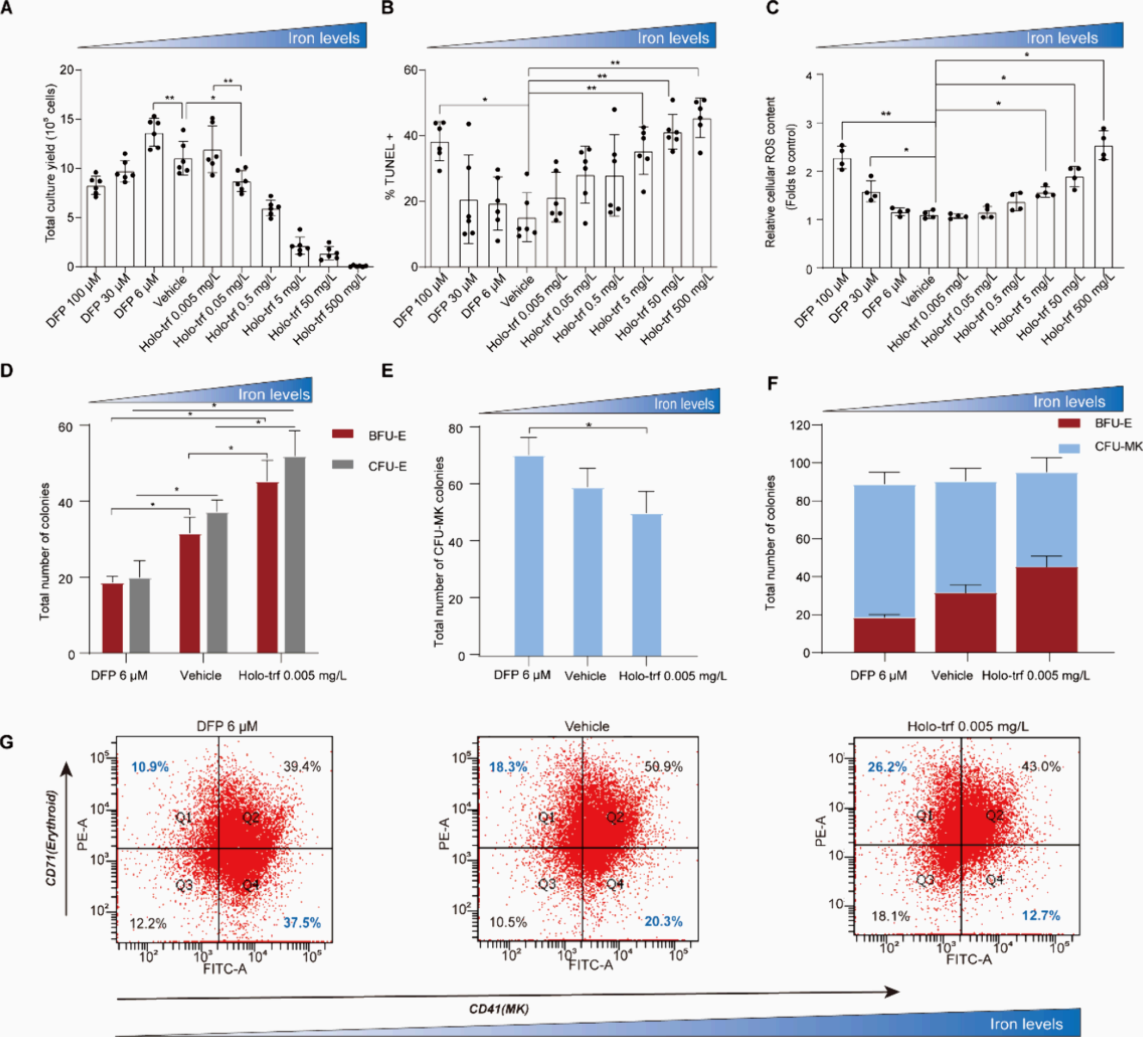
A high concentration of iron drives HSPCs into the erythroid lineage, whereas a low concentration of iron promotes megakaryopoiesis. (A) Absolute numbers of total HSPCs yielded at different iron concentrations on day 5. (B) Detection of apoptosis via the TUNEL assay on day 5. Data are expressed as the mean ± S.D., *n* = 6 in each group. (C) Detection of ROS levels on day 5. Data are expressed as the mean ± S.D., *n* = 4 in each group. Colony counts of burst-forming unit-erythroid (BFU-E), colony-forming unit-erythroid (CFU-E) (D), and colony-forming unit-megakaryocyte (CFU-MK) (E). Data are expressed as the mean ± SD; *n* = 3 in each group. (F) Stacked bar chart of the total number of BFU-E and CFU-MK colonies. (G) Representative images of flow cytometric profiles on day 10. Horizontal axis, CD41 expression; vertical axis, CD71 expression. DFP, deferiprone; Holo-trf, holo-transferrin. *P < 0.05, ** *P* < 0.01.

To elucidate the role of iron on HSPCs’ commitment toward erythroid and megakaryocytic lineages, three different iron concentrations were employed while maintaining a fixed concentration of other cytokines. The result of the CFU assays indicated that the numbers of CFU-E and BFU-E increased with iron concentration increasing in the culturing environment (Figure 1D), while the number of CFU-MK decreased (Figure 1E). The total number of colonies was unchanged (Figure 1F), suggesting that iron drives HSPCs toward the erythroid lineage through competition rather than lineage suppression. The differentiation of HSPCs was further assessed by flow cytometry using the combination of two biomarkers, CD71 and CD41, which are commonly used to characterize erythroid and MK cells, respectively.^9,28^ Consistent with the result of the CFU assay, flow cytometry analysis revealed that the proportion of CD71^+^CD41^–^ cells (erythroid) increased with iron level increases, while the proportion of CD71^–^CD41^+^ cells (MK) decreased with iron addition (Figure 1G, Figure S1). Taken together, these findings indicated that a high concentration of iron drives HSPCs into the erythroid lineage, whereas a low concentration of iron promotes megakaryopoiesis. Additionally, erythroid cells were identified and categorized into distinct populations based on HIS49 and CD71 expression as well as cell size as previously described.^29^ The addition of iron demonstrated an enhancement in the production of erythroid precursor cells in later stages (Figure S2).

### Iron Status Alters the MAPK/ERK Pathway in Rat HSPCs

To better understand how iron availability regulates HSPCs’ commitment into erythroid and megakaryocytic lineages, we analyzed the transcriptome of HSPCs cultured at varying iron concentrations. The results revealed distinct gene expression profiles in HSPCs treated with three different iron levels (Figure S3A, Figure 2A). Specifically, comparing low-iron- to vehicle-treated HSPCs, 108 downregulated and 211 upregulated genes were identified. Similarly, vehicle-treated versus high-iron-treated HSPCs had 446 DEGs, with 62 downregulated and 384 upregulated. Low-iron-treated HSPCs displayed 68 downregulated and 884 upregulated genes compared with high-iron-treated cells (Figure 2A). Over 50% of the DEGs were consistent across all three groups comparisons, with 16 overlapping genes predominantly functioning in cell adhesion, hemopoiesis, cytoskeleton organization, metal ion binding, and regulation of transcription by RNA polymerase (Figure S3B). Consistent with previous reports,^30^ genes related to VEGF and HIF1 were elevated in iron deficiency (Figure S4). Surprisingly, expression levels of transcription factors that govern megakaryopoiesis and erythropoiesis, such as Runx1, Fli1, Tal1, and Myb,^31^ did not differ significantly among different iron concentration groups (Figure S5), suggesting that the transcriptional programs driving these processes are not completely disrupted by iron levels.

**Figure 2:**
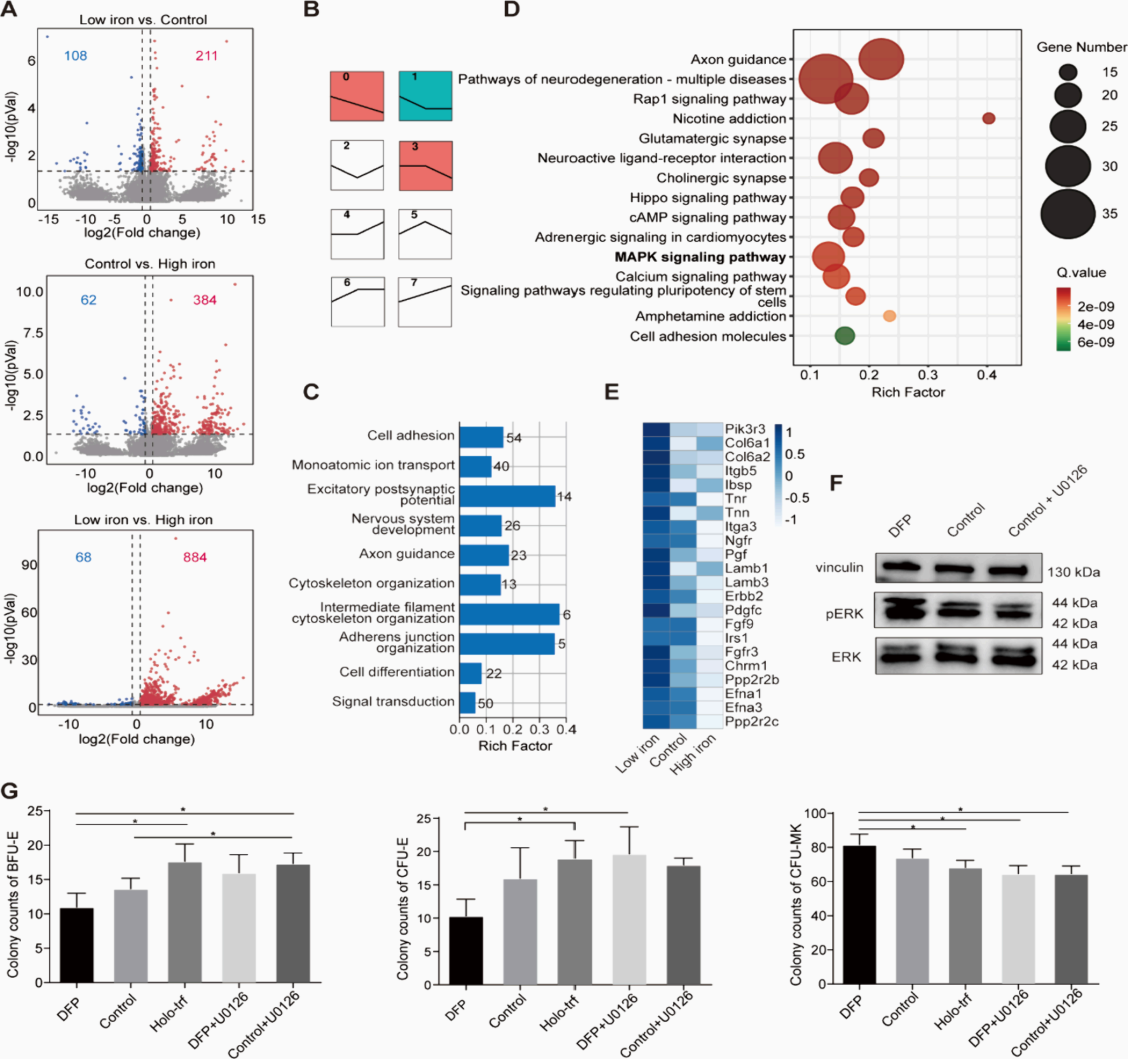
MAPK/ERK pathway acts as a key mediator of iron-regulated HSPC differentiation toward erythroid and megakaryocytic lineages. (A) Volcano plots showing the distribution of gene expression changes in rat HSPCs cultured at three different iron concentrations for 5 days. (B) Genes differently expressed in HSPCs cultured at different iron concentrations were categorized into eight representative profiles with STEM analysis. Significant patterns in STEM analysis were marked with color. Patterns not significant in STEM were white colored. The same color means similar gene expression patterns. (C) Bar chart of GO enrichment analysis for biological process. The *X* axis represents the rich factor, and the *Y* axis represents different GO items. The number beside the bar indicates the number of genes in each GO item. (D) KEGG enrichment analysis. The *X* axis represents the rich factor, while the *Y* axis represents the pathway name. The size of bubble indicates the number of genes in the corresponding pathway. The color of bubble indicates the *Q* value. (E) Heatmap displaying the gene expression profile in the MAPK pathway across different iron concentration groups. (F) Western blot analysis of the protein expression of phospho-ERK1/2 and ERK1/2. (G) Colony counts of BFU-E, CFU-E, and CFU-MK from HSPCs cultured in different iron concentrations in the presence or absence of U0126 for 5 days. Data are expressed as the mean ± SD; *n* = 3 in each group. DFP, deferiprone; Holo-trf, holo-transferrin. * *P* < 0.05.

Subsequently, the 1245 DEGs identified across the three groups were subjected to a STEM analysis, resulting in their categorization into eight representative profiles reflecting their overall expression patterns with increasing iron concentrations (Figure 2B). The majority of DEGs (72.6%) were consistently downregulated (66.7%) or upregulated (5.9%) in response to increasing iron. Genes with biologically relevant and significant profiles were used for KEGG and GO enrichment analyses. A GO enrichment analysis revealed that genes with consistent expression changes with iron levels were associated with cell adhesion, cell differentiation, signal transduction, and cytoskeleton organization (Figure 2C). KEGG pathway analysis showed that DEGs were significantly enriched in the MAPK/ERK pathway (Figure 2D). The literature suggests that MAPK/ERK may control erythroid versus megakaryocytic lineage commitment, as evidenced by its influence on each lineage.^32–36^ For erythropoiesis, ERK pathway blockade in umbilical cord blood mononuclear cells promotes spontaneous erythroid differentiation, resulting in increased numbers of BFU-E colonies and enhanced expression of erythroid glycophorin.^37^ On the contrary, overexpression of the ERK-activating kinase (MAPKK) was observed to inhibit erythroid differentiation, whereas pharmacological inhibition of MAPKK promoted erythroid differentiation.^36^ In the context of megakaryopoiesis,^35^ sustained ERK activation was reported to be required for megakaryocytic differentiation, and persistent activation of the ERK/MAP kinase pathway facilitated the autocrine release of factors that determine megakaryocytic lineage.^34,38^ Consistent with these lines of information, genes encoding relevant proteins in MAPK/ERK pathways were upregulated in HSPCs cultured at a low iron concentration (Figure 2E), which was associated with promoted megakaryopoiesis and inhibited erythropoiesis, as discussed in the preceding section.

### The ERK Pathway Regulates the Functional Output of HSPCs toward Erythroid and Megakaryocytic Lineages

To further validate the role of the ERK pathway on iron-medicated HSPCs’ commitment toward erythroid and megakaryocytic lineages, we evaluated the effect of ERK inhibition on HSPCs’ functional output. Enriched HSPCs were cultured in three different iron concentrations in the presence or absence of U0126, a selective inhibitor of MAPK/ERK kinases.^39^ Consistent with the result in the RNA-seq analysis and previous reports,^40–42^ Western blot data illustrated that phospho-ERK1/2 (Thr202/Tyr204) levels were increased in the low-iron environment, while the introduction of U0126 reduced the expression of phospho-ERK1/2 (Figure 2F, Figure S6). As depicted in Figure 2G, the CFU assay revealed that HSPCs cultured under low-iron conditions produced a significantly lower number of BFU-E and CFU-E compared to cells cultured under high iron levels. In contrast, the number of CFU-MK colonies was significantly higher in the low-iron group. Consistent with the report that the inhibition of ERK signaling by PD0325901 results in the spontaneous erythroid differentiation of umbilical cord blood mononuclear cells,^37^ the U0126-treated group showed a significant increase in BFU-E colonies when compared to those in the control group. In contrast, the number of CFU-MK colonies decreased with U0126 treatment, although this was not statistically significant. Of note, when comparing the group of low iron with group treated with “low iron + U0126”, the inhibition of the ERK pathway with U0126 completely aborted the effect of low iron on promoting CFU-MK and inhibiting BFU-E and CFU-E growth. Collectively, these results suggested that iron may regulate erythroid versus megakaryocytic lineages through the MAPK/ERK pathway

### Iron Deficiency is Associated with Erythrocytopenia and Thrombocytosis in Rats

To further explore the impact of iron on erythroid and megakaryocytic lineages *in vivo*, we induced IDA in rats by maintaining an iron deficiency diet during the whole course of experiment and phlebotomizing twice a week for the initial 3 weeks (Figure 3A). Five weeks after model development, rats with IDA exhibited significantly decreased HGB, mean corpuscular volume (MCV), mean corpuscular hemoglobin (MCH), and hematocrit (HCT) when compared with healthy controls (Figure 3B). Furthermore, IDA rats demonstrated markedly lower serum iron concentrations and ferritin levels compared to the healthy controls, suggesting that the IDA model was successfully established (Figure 3C). Corroborating findings from an *in vitro* study, rats with iron deficiency displayed significantly elevated PLT counts and decreased RBC compared to their healthy counterparts (Figure 3D).

**Figure 3:**
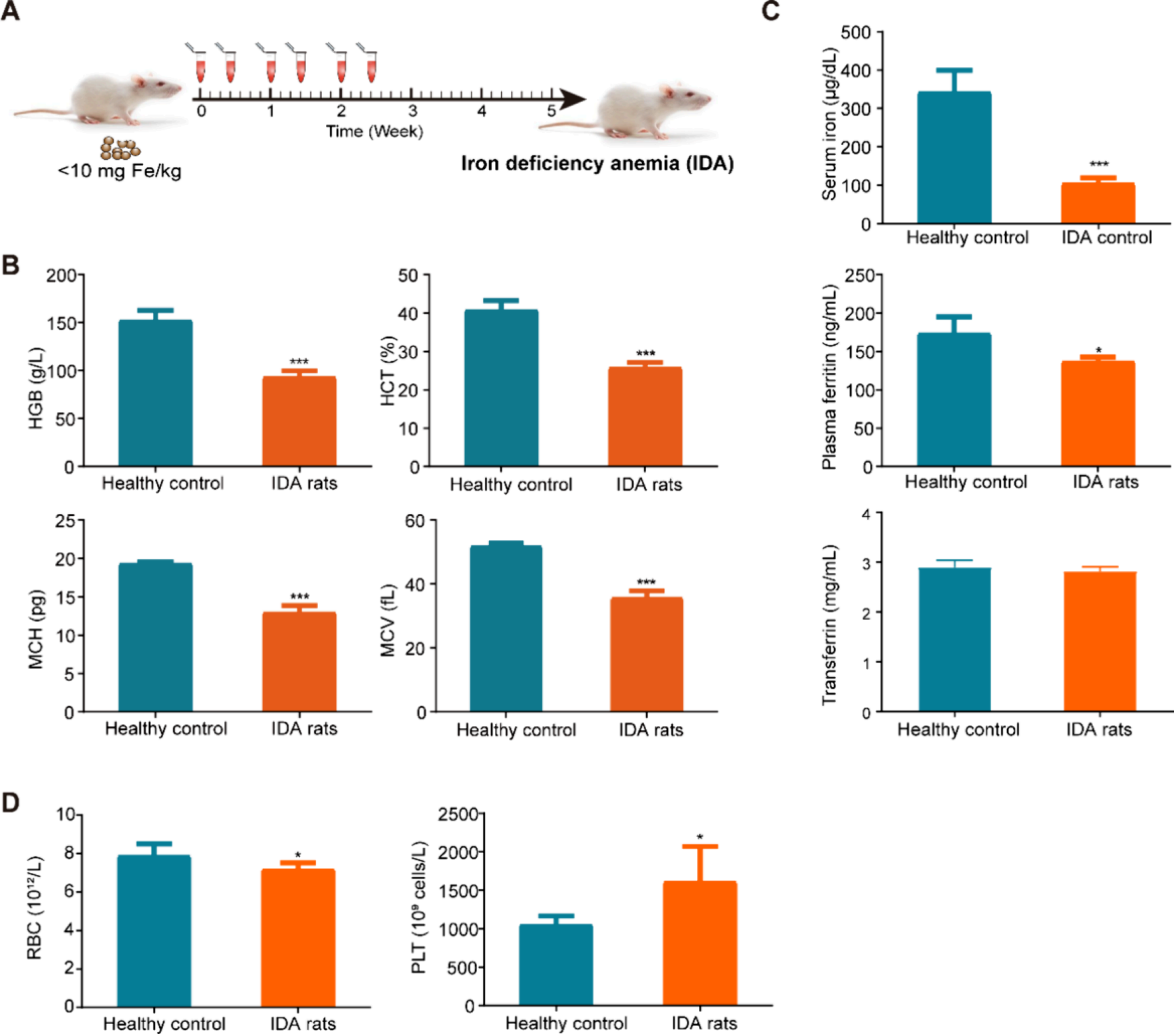
Iron deficiency is associated with erythrocytopenia and thrombocytosis. (A) Diagram of developing the iron deficiency anemia (IDA) model in SD rats. (B–D) Hematological parameters and parameters for iron status assessment in healthy control (*n* = 3) and IDA rats (*n* = 24) on the end point of the IDA model establishment (day 37). Data were expressed as the mean ± SD. *P < 0.05, **P < 0.01, ***P < 0.001. HGB, hemoglobin; HCT, hematocrit; MCV, mean corpuscular volume; MCH, mean corpuscular hemoglobin; RBC, red blood cell count; PLT, platelet count.

### Intravenous Iron Supplement Rescues Thrombocytosis and Increases RBC in IDA Rats

To examine the effects of different iron levels on erythropoiesis and thrombopoiesis *in vivo*, a PK/PD study of intravenous iron was conducted in IDA rats. IDA rats were administered with three different doses (3, 15, and 90 mg/kg) of iron intravenously twice a week for 2 weeks (Figure 4A). A great drop in iron concentration in serum was observed 48 h after dosing (Figure 4B). This indicated a rapid distribution of intravenous iron from blood to peripheral tissues, as the body lacks a regulated mechanism for iron excretion.^43^ HGB concentration demonstrated relative stabilization with the progression of iron deficiency, which may be attributed to the fact that the HGB concentration had reached the ultimate level for sustaining normal life activities with mobilization of stored iron to support HGB synthesis. Intravenous iron supplementation led to a dose-dependent increase in HGB levels (Figure 4C).

**Figure 4:**
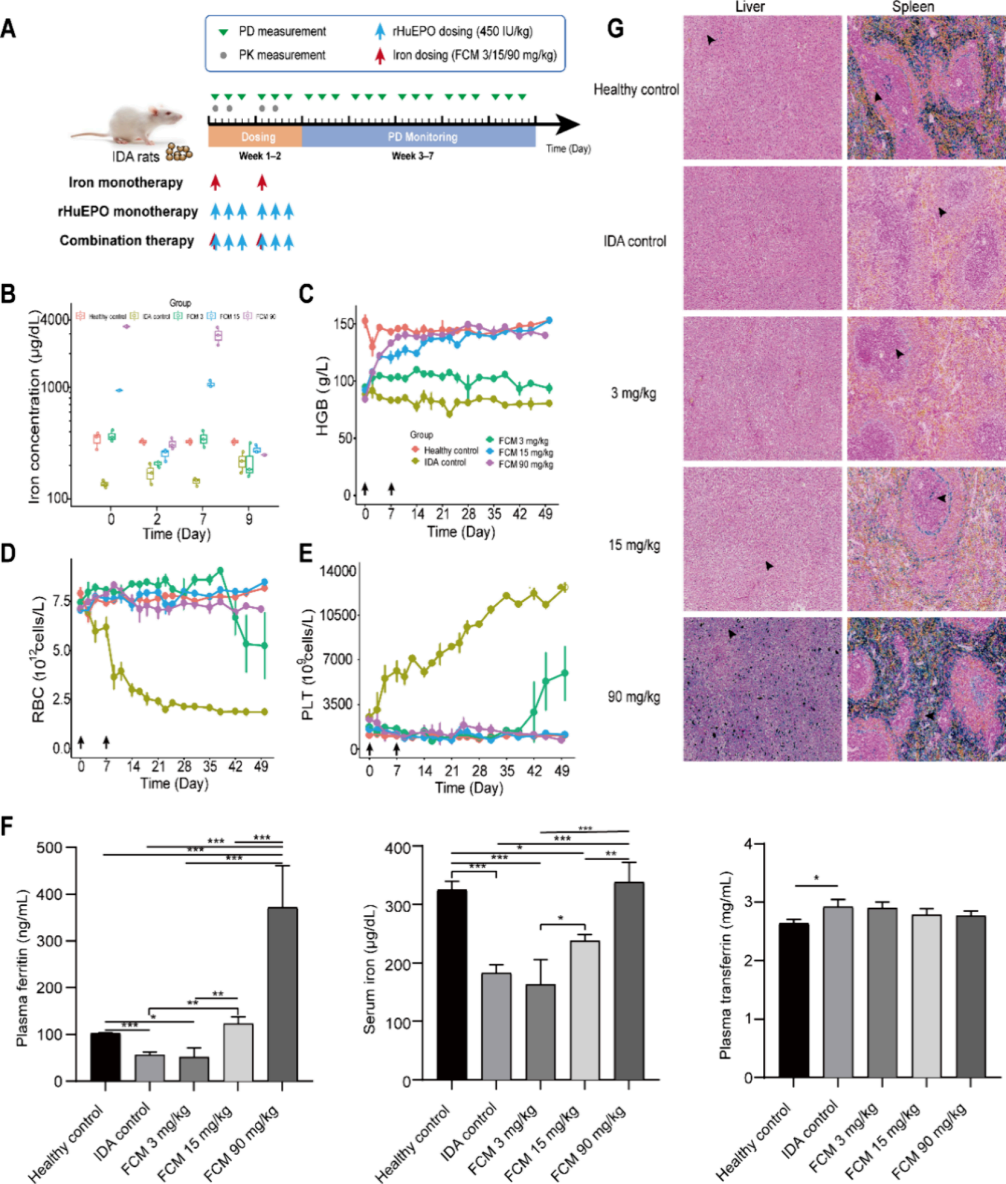
Iron supplement rescues the thrombocytosis and increases RBC in IDA rats. (A) Schematic representation of the pharmacokinetic (PK) and pharmacodynamic (PD) study design in IDA rats. (B) Iron concentrations after first dosing in different treatment groups. (C–E) Hematological parameters versus time in healthy controls, IDA controls, and IDA rats treated with FCM at the dosage of 3, 15, or 90 mg/kg once weekly for 2 weeks. Data are expressed as the mean ± SE with *n* = 3 in each group. The black arrows in the line charts represent the dosing events of FCM. (F) Parameters for iron status assessment at the end point (7 weeks after first dosing). (G) Liver and spleen sections stained with Prussian blue for iron detection. The blue area corresponds to iron staining (black arrows). Different treatment groups are shown perpendicularly. The results shown are from one representative experiment and one representative animal per group. Scale bar: 50 μm.

With the progression of iron deficiency, IDA rats exhibited a continuous decline of RBC and elevation of PLT counts, while iron supplementation increased RBC counts and inverted the escalating trend of PLT (Figure 4D,E). Notably, unlike the dose-dependent effect of iron on HGB production, three different doses initially had a relatively equivalent effect on increasing RBC counts and decreasing PLT counts. This suggests a distinct mechanism of iron action in promoting RBC production compared with its effect on HGB synthesis. Decreased RBC and elevated PLT were observed in rats administered with 3 mg/kg iron 5 weeks after the first dosing. It may be explained that the dose of 3 mg/kg QW for 2 weeks could not rescue iron deficiency in the rats, which eventually led to decreased RBC and elevated PLT, just like those in the IDA control group. This was supported by the significantly decreased serum iron concentration and plasma ferritin level together with lower iron content in the liver and spleen in the 3 mg/kg treatment group when compared to the healthy controls and the other two treatment groups (Figure 4F,G).

### EPO Enhanced the Effect of Iron on Erythropoiesis and Thrombopoiesis

Given the common clinical practice of coadministering ESA and intravenous iron agents, the impact of rHuEPO on iron-regulated erythropoiesis and thrombopoiesis was evaluated. Initially, the effect of rHuEPO was investigated *in vitro* using a CFU assay. HSPCs were cultured at different combination groups of rHuEPO and iron concentrations. Consistent with the result at EPO 1 IU/mL (Figure 1D,E), iron promoted the number of BFU-E and CFU-E and inhibited the growth of CFU-MK in a dose-dependent manner when the rHuEPO concentration was fixed at 10 IU/mL (Figure 5A,B). Furthermore, when comparing different rHuEPO concentration groups, the addition of rHuEPO was found to enhance the effect of iron on thrombopoiesis and erythropoiesis.

**Figure 5:**
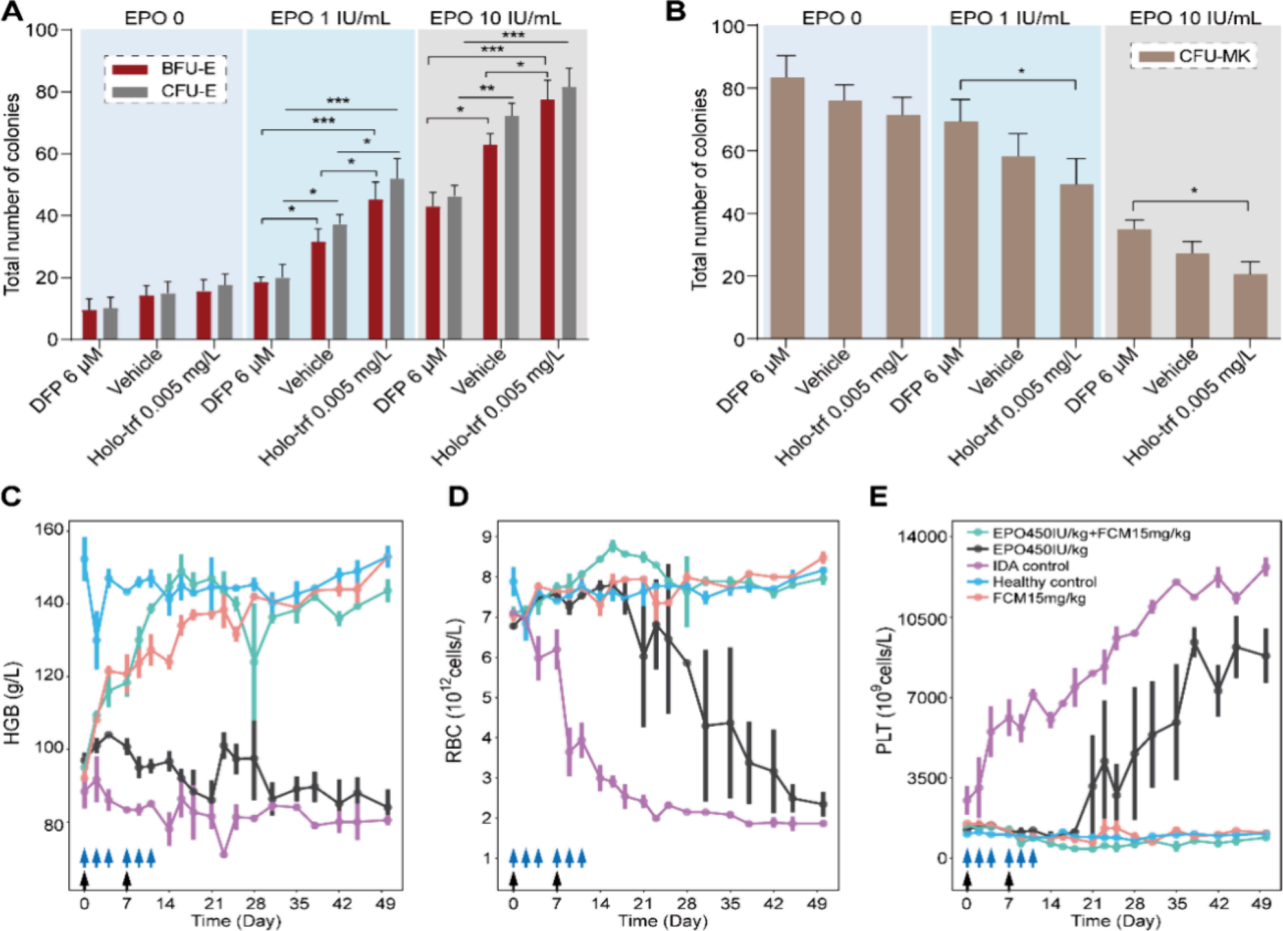
EPO enhanced the effect of iron on erythropoiesis and thrombopoiesis. (A/B) Colony counts of burst-forming unit-erythroid (BFU-E), colony-forming unit-erythroid (CFU-E) (A), and colony-forming unit-megakaryocyte (CFU-MK) (B) at different combinations of iron and EPO concentrations. DFP, deferiprone; Holo-trf, holo-transferrin. Data are expressed as mean ± SD; *n* = 3 in each group. *P < 0.05, **P < 0.01, ***P < 0.001. (C–E) Hematological parameters, including hemoglobin (HGB, C), red blood cell count (RBC, D), and platelet count (PLT, E), in IDA rats treated with saline (IDA control group), iron monotherapy (15 mg/kg, QW for 2 weeks), rHuEPO monotherapy (450 IU/kg, TIW for 2 weeks), or a combination of iron and rHuEPO. Data are expressed as the mean ± SE; *n* = 3 in each group. The black arrows in the line charts represent dosing events of iron, while the blue arrows in the line charts represent dosing of rHuEPO.

The effect of rHuEPO on iron-regulated erythropoiesis and thrombopoiesis was further explored *in vivo*. IDA rats were given rHuEPO monotherapy, iron monotherapy (15 mg/kg), or a combination of rHuEPO and iron (Figure 4A). As depicted in Figure 5C, rHuEPO monotherapy gave rise to a negligible increase in HGB production in IDA rats. Although they slightly increased RBC levels at the early stage of treatment, IDA rats treated with rHuEPO monotherapy still exhibited declined RBC and elevated PLT with the progression of iron deficiency (Figure 5D,E). Nevertheless, the group treated with the combination therapy of rHuEPO and iron showed significantly higher HGB and RBC levels compared to those treated with iron monotherapy (Figure 5C,D). Notably, although EPO is known for stimulating erythropoiesis, a combination with rHuEPO enhanced the effect of iron supplementation on decreasing PLT count (Figure 5E).

### Quantitative Analysis of the Iron-Regulated HSPCs’ Commitment toward Erythroid and Megakaryocytic Lineages through a Mechanism-Based PK/PD Model

To quantify the impact of iron on erythropoiesis and thrombopoiesis, a mechanism-based PK/PD model was developed. The model structure is illustrated in Figure 6. A sequential modeling approach was employed in which the iron concentrations from the PK model drove changes of hematological parameters in the PD model. As the visual predictive check plot shows in Figure S7, the proposed PK model effectively captures the profiles of serum iron concentrations with adequate precision of the parameters (Table S2). Additionally, the developed PD model accurately depicts the dynamics of RBC, PLT, and HGB levels in both IDA control rats and IDA rats given different doses of iron treatments (Figure 7A). No significant structural bias or obvious systemic deviations were found in the final model (Figures S8 and S9).

**Figure 6:**
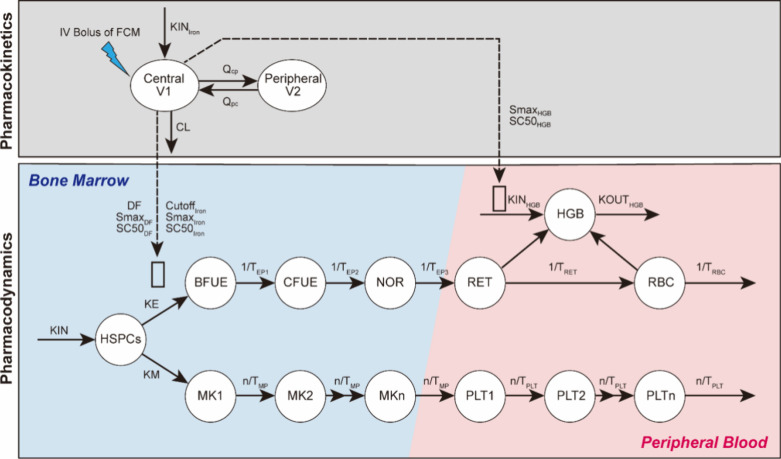
Schematic diagrams of the mechanism-based PK/PD model for the effect of iron on RBC, HGB, and PLT production. KIN_Iron_ is a zero-order input rate constant of iron. V1 and V2 denote the volumes of the central and peripheral compartments of iron, respectively. *Q*_CP_ and *Q*_PC_ are the clearances from the central compartment to the peripheral compartment and from the peripheral compartment to the central compartment, respectively. CL is the clearance of iron. KIN is the zero-order rate constant for producing HSPCs. KE and KM are the first-order rate constant for HSPCs to differentiate into erythroid and MK lineages, respectively. *T*_EP_ represents the average time required for precursors to develop into the next cell population. *T*_RET_ and *T*_RBC_ represent the mean residence times for reticulocytes (RETs) and RBC, respectively. The series of *n* = 10 aging compartments (MK*i*, *i* = 1, ..., *n*) denotes the MK precursor cells, with the first-order transition rates *n*/*T*_MP_; PLT*i* (*i* = 1, ..., *n*) represents the platelets with the transition rates *n*/*T*_PLT_. The open rectangle indicates the effects of iron. Smax_HGB_ denotes the maximal stimulus of iron on HGB production, and SC50_HGB_ is the change of iron concentration from baseline that induces a half-maximum effect of HGB production. DF is a disease progression factor in IDA rats. Smax_DF_ is the maximal correction of DF after intravenous iron supplement. SC50_DF_ is the change of iron concentration from the baseline that induces a half-maximum effect on DF correction. Smax_Iron_ is the maximal stimulus of iron on promoting HSPCs toward BFU-E, and SC50_Iron_ is the change of iron concentration from baseline that induces a half-maximum effect. HSPCs, hematopoietic stem and progenitor cells; BFUE, burst-forming unit-erythroid cells; CFUE, colony-forming unit-erythroid cells; NOR, normoblasts.

**Figure 7:**
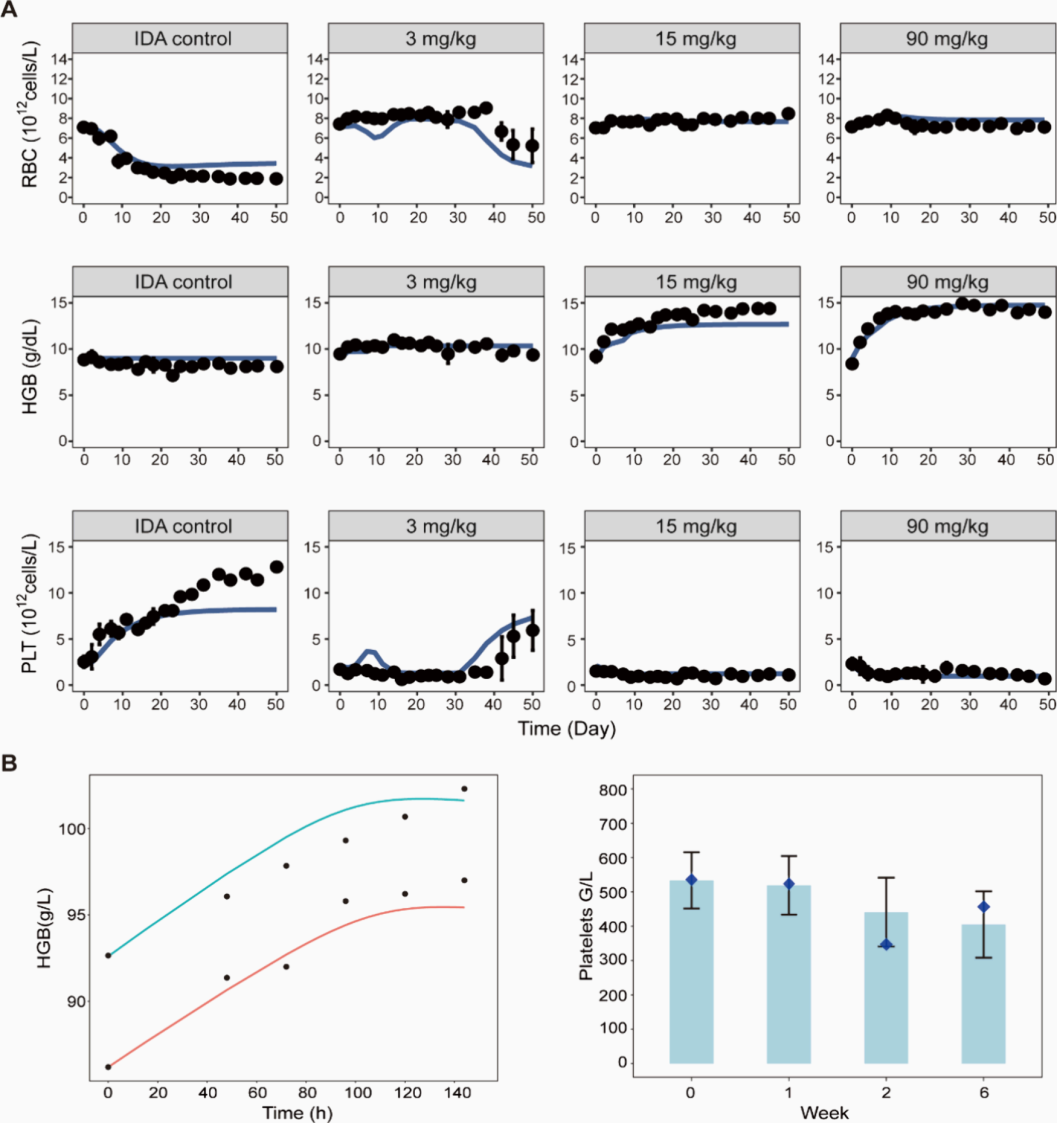
Evaluation of the mechanism-based PK/PD model in rats and humans. (A) Red blood cell (RBC), hemoglobin (HGB), and platelet (PLT) levels versus time profile in the IDA control group and different treatment groups. Symbols depict mean ± SD (*n* = 3 in each group), whereas the solid lines represent model prediction. (B) Validation of the model with human data. In the left figure, the blue and red lines represent the model predicted profile of HGB versus time in patients with IDA after a single dose of 500 and 1000 mg FCM, respectively. The black dots are observed data in humans. In the right figure, the bar chart is the reported data in human after administration of 500 mg FCM for continuous 3 weeks, while the points in blue diamond shapes denote model predicted values.

Parameter estimates of the final PD model are shown in Table 1, while secondary parameter calculations based on baseline conditions and estimations are given in Table S3. The estimated values of the lineage-related parameters including *T*_RET_, *T*_PLT_, RBC_0_, HBG_0_, and PLT_0_ were close to physiologic values.^3,6,44^ The estimated RBC lifespan in the model is 168.4 h, which is greatly shorter than that in normal rats. It is reasonable because iron deficiency was reported to reduce the lifespan of RBC.^22^ The disease factor, DF, was estimated to be 0.87 in the final model, indicating an 87% decrease in the differentiation rate of HSPCs into erythroid lineage when compared to healthy conditions. An iron concentration exceeding the cutoff value of 201.284 μg/dL promotes HSPCs toward BFU-E with Smax and SC_50_ estimated to be 12.63 and 3.14 μg/dL, respectively. Additionally, iron stimulates HGB production via an Emax function, with Smax and SC_50_ values estimated at 0.72 and 16.24 μg/dL, respectively. All parameters were estimated with reasonable precision with relative standard errors (RSE%) below 38.93%. Model-based simulations enabled a clear depiction of the dynamic process of erythroid and megakaryocytic progenitors under varying iron concentrations. In IDA control rats, BFU-E experienced a continuous decline with iron deficiency progression before stabilizing at around 45% of baseline levels, while MK progenitor numbers increased and stabilized at a plateau 3 times higher than the baseline (Figure S10). Conversely, iron supplementation promotes BFU-E and suppresses MK, consequentially leading to the observed increase in RBC counts and decline in PLT levels.

**Table 1:** Parameter Estimates in the Final Pharmacodynamic Model

parameters	definition	units	estimate	% RSE
*T*_ERT_	mean residence time for RETs	h	39.92	16.27
*T*_RBC_	mean residence time for RBCs	h	161.8	14.06
RBC_0_	baseline of RBC counts	×10^12^ cells/L	7.141	5.25
DF	disease progression factor on HSPCs' differentiation into BFU-E		0.87	4.94
Smax_Iron_	maximal stimulus of iron on HSPCs' differentiation into BFU-E		12.63	32.24
SC50_Iron_	change of iron concentration from baseline that induces a half-maximum effect	μg/dL	3.14	38.93
Smax_HGB_	maximal stimulus of iron on the production of HGB (KIN_HGB_)		0.72	25.3
SC50_HGB_	change of iron concentration from baseline that induces a half-maximum effect on KIN_HGB_	μg/dL	16.24	37.73
HGB_0_	baseline HGB concentration	g/dL	8.99	4.94
*T*_MP_	mean lifespan of megakaryocytic precursor cells	h	3.49	38.5
*T*_PLT_	mean lifespan of platelets	h	85.7	18.79
PLT_0_	baseline platelets count in blood	×10^12^ cells/L	2.12	18.67
KE	the first-order rate constant for differentiation of HSPCs into BFU-E	×10^–4^/h	166.5	16.22
Cutoff_Iron_	cutoff of iron concentration change from baseline to stimulate HSPCs' differentiation into BFU-E	μg/dL	4.28	16.72
SC50_DF_	SC50 of intravenous iron on correction of disease progression	μg/dL	2062	7
σ_prop‑RBC_	proportional residual error of RBC		0.29	7.13
σ_add‑HBG_	additive residual error of HGB		0.79	5.11
σ_prop‑PLT_	proportional residual error of PLT		0.58	6.80

### Extrapolation of the Model to Predict Erythropoiesis and Thrombopoiesis in Humans

To demonstrate the capacity of our model in characterizing the effect of iron on erythropoiesis and thrombopoiesis in humans, we extrapolated the model to humans and validated the model with published clinical data.^24,25^ Given the availability of sufficient serum iron concentration data in the literature,^20^ the PK model was directly refitted with data in patients with IDA (Figure S11). The PD model was adapted for humans by translating the parameters using physiological values that are specific to humans. The adapted model was then used to simulate the dynamics of HGB and PLT counts under various intravenous iron treatments in patients with IDA. As shown in Figure 7B, a good agreement was found between our model predictions and reported data in humans.^24,25^ This provides compelling proof of concept for the capacity of our model to accurately recapitulate the effects of iron on erythropoiesis and thrombopoiesis in humans, highlighting its clinical utility in future work for guiding the dose optimization of iron therapy.

## Discussion

Iron is a crucial micronutrient for nearly all living organisms, as it is important for numerous biological processes, including oxygen transport, DNA synthesis, and electron transport.^45^ In hematopoiesis, although the significance of iron in erythrocyte hemoglobinization is well recognized, there remains a need to investigate its influence on other facets of blood cell biology. Recent studies have revealed that iron acts as a critical regulator of the fate of adult hematopoietic stem cells, influencing long-term maintenance and preventing dysfunction associated with aging.^46^ Xavier-Ferrucio et al. demonstrated that a lack of iron favored megakaryocytic commitment of MEP.^11^ However, the role of iron in directing HSPCs toward erythroid and megakaryocytic lineages remains controversial due to the lack of supporting data from critical erythroid lineage and concerns about the rationale for using transferrin receptor 2 (TfR2) knockdown to mimic low-iron status in their study.^47^ Furthermore, it is indispensable to quantitatively analyze its effect on these two lineages for elucidating iron’s role in erythropoiesis and thrombopoiesis development. This study aims to clarify the pivotal role of iron in directing HSPCs’ commitment toward erythroid and megakaryocytic lineages through both experimental and mathematical approaches.

Using CFU assays, we demonstrated that increasing iron availability in the culture environment led to an increase in the number of CFU-E and BFU-E, while the count of CFU-MK decreased. The total number of colonies remained unchanged, suggesting that iron drives HSPCs toward the erythroid lineage through competition rather than lineage suppression. Our flow cytometry analysis aligned with the CFU assays, showing that iron supplementation in the HSPC culture environment enhanced erythroid cell proportions and restrained differentiation into the megakaryocytic lineage.

We further demonstrated with transcriptomic and functional output analysis that iron regulates the HSPCs’ commitment into the two lineages through the MAPK/ERK pathway. The association between low iron and upregulated phospho-ERK was previously reported in lymphocytes and THP-1 cells.^48,49^ In addition, defective TfR2 has been linked to iron overload,^41^ with mice deficient in TfR2 exhibiting reduced phospho-ERK.^41,42^ Consistently, our study revealed an upregulated MAPK/ERK pathway in HSPCs cultured under low-iron conditions.

After the *in vitro* studies, we continue to demonstrate the impact of iron on erythropoiesis and thrombopoiesis *in vivo.* We chose rats over mice for several reasons. First, rats allow for more feasible serial blood sampling for pharmacokinetic and hematological parameters evaluation due to their larger blood volume, approximately 8-fold greater than that in mice.^50^ This not only reduces the number of animals needed but also enhances data reliability by minimizing interanimal variability. Second, despite the widespread use of mouse models owing to the ease of obtaining genetically modified animals and more well-defined markers, research suggests that rat erythropoiesis is more similar to that of humans, particularly regarding spleen function in both steady-state and stress conditions.^7^ The spleen is a crucial organ in mice for adult erythropoiesis, especially under stress.^51^ In mice, extramedullary erythropoiesis, indicated by splenomegaly, compensates for anemia, often obscuring significant phenotypes.

Taken together, our *in vitro* and *in vivo* studies demonstrate that iron plays a vital role in directing HSPCs’ commitment toward erythroid and megakaryocytic lineages. A high concentration of iron drives HSPCs into the erythroid lineage, whereas a low concentration favors megakaryocytopoiesis. This effect of iron was enhanced in combination with EPO. Nevertheless, it should be noted that this conclusion does not apply to extreme cases of severe iron deficiency or iron overload. When iron levels are severely depleted, hematopoiesis is impaired, leading to both thrombocytopenia and erythrocytopenia.^52^ Conversely, excessive iron levels can impair bone marrow function, lead to mitochondrial damage related to ferroptosis, and decrease the number of HSPCs.^53^ With genetic or acquired iron overload, some individuals may experience thrombocytopenia owing to the impact of iron overload on the bone marrow, while others may have a normal or even elevated platelet count.^54,55^ This variability may arise because hematological parameters can also be complicated by other factors such as concurrent conditions or complications.^47^ In addition, a limitation of our study is the lack of direct validation of these findings using human-derived HSPCs. While our experiments were conducted in well-established rat models, widely recognized for their genetic and physiological similarities to humans in hematopoiesis, we acknowledge the possibility of species-specific differences. Future studies involving human-derived HSPCs are needed to provide further validation and expand upon our findings.

A PK/PD model was developed to quantify the effect of iron on the two lineages. This model incorporates a fundamental physiological structure and effectively captures the dynamics of erythropoiesis and thrombopoiesis under different iron conditions in both rats and humans. In recent years, the thrombotic risk linked to thrombocytosis in iron deficiency has gained recognition.^5,6^ Patients undergoing iron therapy for conditions like chronic kidney disease or heart failure often exhibit platelet abnormalities and significantly increased risk of thromboembolic complications.^56^ Given our findings regarding the role of iron in erythropoiesis and thrombopoiesis, platelets should be considered as an important PD marker in iron therapy rather than solely evaluating HGB levels. This translational study bridges mechanistic insights into the regulatory role of iron on HSPC commitment to erythroid and megakaryocytic lineages with the clinical utility of intravenous iron therapy through mechanism-based PK/PD modeling. Notably, according to the established model, a dose of 90 mg/kg has a negligible additional increase in terms of the stimulation effect on both HGB production and PLT inhibition compared with a 15 mg/kg dose. However, significant iron accumulation was observed in the dose of 90 mg/kg, accompanied by multifocal lymphocytic infiltrate (Figure 4G, Figure S12). Iron overload is linked to tissue damage, an increased risk of infection, and tumor progression.^57^ Therefore, optimal dose selection could be achieved using a modeling and simulation approach to attain the desired HGB target for anemia treatment, maintain platelet levels within the physiological range to reduce thrombosis risk, and minimize iron accumulation in organs to mitigate adverse effects.

In conclusion, integrating biological and computational approaches, this study elucidated the pivotal role of iron on HSPCs’ commitment toward erythroid and megakaryocytic lineages. Our *in vitro* and *in vivo* findings revealed that a high concentration of iron drives HSPCs into the erythroid lineage and inhibits differentiation into the megakaryocytic lineage, whereas a low concentration of iron promotes the megakaryocytic lineage. We demonstrated that this effect of iron is mediated by the MAPK/ERK pathway and is potentiated in combination with EPO. Furthermore, a mechanism-based PK/PD model was developed to quantify the effect of iron on the two lineages. The dynamic interplay between iron levels and the development of erythropoiesis and thrombopoiesis was accurately recapitulated in both rats and humans. Overall, this work not only provides functional insights into the pivotal role of iron on erythropoiesis and thrombopoiesis but also lays the groundwork for optimizing iron therapy in anemia treatment and potentially other hematologic conditions where erythropoiesis and thrombopoiesis are affected.

## Supplementary Material



## Notes

Supporting information and methods on animal study, *in vitro* study, flow cytometric analysis, bioassays and hematological measurements, Prussian blue staining, and Western blot analysis; statistical analyses plots of CD71^+^CD41^–^ cells and CD71^–^CD41^+^ cells in flow cytometry; flow cytometry analysis of erythroid cells; heatmap and Venn diagram in RNA-seq; the mRNA expression of DEG related to VEGF and HIF1; the mRNA expression of genes of transcription factors known to regulate megakaryopoiesis and erythropoiesis; normalized phospho-ERK and ERK levels; serum iron concentration versus time profiles in rats; goodness-of-fit plots of the final PK model; goodness-of-fit plots of the final PD model; model-based simulation of the dynamic process of the BFU-E and MK compartment; serum iron concentration versus time profiles in patients with IDA; HE staining of the liver, heart, kidney, and spleen; parameters in humans for the adapted PD model; parameter estimates obtained from the PK model; and calculation of the secondary parameters based on the baseline condition and PD model estimation (PDF)

## Notes

Address: Eighth Floor, Lo Kwee-Seong Integrated Biomedical Sciences Building, Area 39, The Chinese University of Hong Kong, Shatin, N.T. Hong Kong

## Abbreviations

HSPCs: hematopoietic stem and progenitor cells
HGB: hemoglobin
RBC: red blood cell
PLT: platelet
ESA: erythropoiesis-stimulating agent
EPO: erythropoietin
TfR2: transferrin receptor 2
TPO: thrombopoietin
IDA: iron deficiency anemia
MEP: megakaryocytic-erythroid progenitor
PK: pharmacokinetic
PD: pharmacodynamic
DFP: deferiprone
rHuEPO: recombinant human erythropoietin
GM-CSF: granulocyte-macrophage colony-stimulating factor
FCM: ferric carboxymaltose
BFU-E: burst forming unit-erythroid cells
CFU-E: colony-forming unit-erythroid
CFU-MK: colony-forming unit-megakaryocyte
Holo-trf: holo-transferrin
MK: megakaryocytic
NORs: normoblasts
RETs: reticulocytes
STEM: Short Time-series Expression Miner
QW: once weekly
TIW: thrice weekly
DEGs: differentially expressed genes
GO: Gene Ontology
KEGG: Kyoto Encyclopedia of Genes and Genomes
HCT: hematocrit
MCV: mean corpuscular volume
MCH: mean corpuscular hemoglobin
ERFE: erythroferrone
PHD2: prolyl hydroxylase 2
SD: Sprague–Dawley
IMDM: Iscove’s modified Dulbecco’s medium
