# Supplementary material for: Mechanism-Based Pharmacokinetic/Pharmacodynamic Modeling for Iron-Regulated Hematopoietic Stem and Progenitor Cells’ Commitment toward Erythroid and Megakaryocytic Lineages
Source: ACS Pharmacol Transl Sci. 2025 May 30;8(6):1711–25. doi: 10.1021/acsptsci.5c00097 (PMC12171892; doi:10.1021/acsptsci.5c00097)
Supplement: Supplementary file 1 [file pt5c00097_si_001.pdf]

Mechanism-based pharmacokinetic/pharmacodynamic modeling for iron-regulated hematopoietic stem and progenitor cells' commitment towards erythroid and megakaryocytic lineages

Kangna Cao[a,], Xiaoqing Fan[a,], Raymond S. M. Wong[b], Xiaoyu Yan[a,*]

[a] Guangdong-Hong Kong-Macao Joint Laboratory for New Drug Screening, School of Pharmacy, The Chinese University of Hong Kong, Hong Kong SAR, P. R. China

[b] Division of Hematology, Department of Medicine and Therapeutics, Faculty of Medicine, The Chinese University of Hong Kong, Hong Kong SAR, P. R. China

[*]Corresponding author: Xiaoyu Yan, PhD (E-mail: xiaoyuyan@cuhk.edu.hk)

Number of pages: 16

Number of figures: 12

Number of tables: 3

Table of Content

Category	Title	Page
Supplemental material and methods	Animals	S2
	Proliferation and differentiation of rat HSPCs	S3
	Flow cytometric analysis	S4
	Bioassays and hematological measurements	S4
	Prussian blue staining	S5
	Western blot analysis	S5
Figures	Figure S1. Comparison of the proportion of CD71+CD41- cells (erythroid, A) and CD71-CD41+ cells (MK, B) in HSPCs treated with three different iron concentrations using flow cytometry.	S7
	Figure S2. Flow cytometry analysis of erythroid cells.	S8
	Figure S3. Distinct gene expression profiles in HSPCs treated with three different iron levels.	S9

Category	Title	Page
	Figure S4. mRNA expression of differently expressed genes related to VEGF(A) and HIF1 (B) in HSPCs cultured at different iron concentrations.	S9
	Figure S5. mRNA expression of genes of transcription factors known to regulate megakaryopoiesis and erythropoiesis, including Runx1, Fli1, Tal1, and Myb.	S10
	Figure S6. Normalized phospho-ERK (A) and ERK levels (B) in Western blot analysis.	S10
	Figure S7. Serum iron concentration versus time profiles in rats.	S11
	Figure S8. Goodness-of-fit plots of the final PK model.	S11
	Figure S9. Goodness-of-fit plots of the final PD model.	S12
	Figure S10. Model-based simulation of the dynamic process of the BFU-E (A) and MK (B) compartment.	S12
	Figure S11. Serum iron concentrations versus time profiles in patients with IDA.	S13
	Figure S12. Liver, heart, kidney and spleen sections underwent hematoxylin-eosin (HE) staining.	S14
Tables	Table S1. Parameters in humans for the adapted PD model.	S15
	Table S2. Parameter estimates obtained from the PK model.	S15
	Table S3. Calculation of the secondary parameters based on the baseline condition and PD model estimation.	S16

Supplemental material and methods

Animals

Male Sprague-Dawley (SD) rats were utilized due to the similarities between rats and humans in erythropoiesis.[8] Rats weighing between 160 to 200 grams were acquired from the Laboratory Animal Services Centre in the Chinese University of Hong Kong and were acclimatized for at least one week prior to the experiment. The animals were housed in a controlled environment with unlimited access to water and food. To induce IDA, the rats were maintained on a low iron diet (<10 mg Fe/kg) throughout the experiment. Additionally, the rats underwent bi-weekly phlebotomy, with 1 mL of blood withdrawn each time during the first three weeks, followed by a two-week stabilization period. Healthy control animals were provided with a normal iron diet (200

mg Fe/kg) without phlebotomy. Hematological parameters were monitored twice weekly throughout the five-week period of model establishment.

Proliferation and differentiation of rat HSPCs

Enriched HSPCs from SD rats were employed to create an *in vitro* system for examining the effects of varying iron concentrations, both alone and in combination with EPO, on the proliferation and differentiation of HSPCs. Rats were euthanized under isoflurane anesthesia through exsanguination, followed by the removal of femurs and extraction of bone marrow using Iscove's Modified Dulbecco's Medium (IMDM). The cells were then collected into a centrifuge tube of 15 mL with 3 mL of Ficoll-Paque PLUS (GE17-1440-02, Merck, Darmstadt, Germany) and centrifuged at 645 g for 20 minutes at 4°C. After centrifugation, with the upper layers discarded, the retaining buffy coat was then washed with IMDM. Following another centrifugation, the cells were resuspended in IMDM. Enrichment of HSPCs was achieved using the EasySep™ Rat Custom Positive Selection Kit (STEMCELL Technologies, Vancouver, Canada) and mouse anti-rat CD90 (BD Biosciences, San Jose, CA, USA). The enriched cells were cultured in StemSpan™ SFEM (STEMCELL) at 37°C with 5% CO_2.

The culture process was divided into two sequential steps. During the first step (days 0–5), HSPC proliferation was induced by adding 100 ng/mL fms-related tyrosine kinase 3 ligand (FL; STEMCELL), 50 ng/mL TPO (TPIAO; Shenyang Sunshine Pharmaceutical Co., Shenyang, China), 100 ng/mL stem cell factor (SCF; R&D Systems, MN, USA), and varying concentrations of iron. As the medium in the control group contained holo-transferrin, different iron concentrations were achieved by adding varying amounts of additional holo-transferrin (0.005, 0.05, 0.5, 5, 50, 500 mg/L; Sigma-Aldrich, MO, USA) or the iron chelator deferiprone (DFP; 6, 30, and 100 μM; MedChemExpress, Shanghai, China). On day 5, cells were stained with trypan blue and counted. Quantitative detection of apoptosis was performed using a TUNEL assay with the TiterTACS Kit (R&D Systems, MN, USA). Reactive oxygen species (ROS) level was measured with Cellular ROS Assay Kit (ab113851, Abcam Limited, Cambridge,

UK). To assess the impact of iron and EPO on HSPCs differentiation, various concentrations of iron (DFP 6 μM, vehicle control, holo-transferrin 0.005 mg/L) and recombinant human erythropoietin (rHuEPO; vehicle control, 1 IU/mL, 10 IU/mL) were applied during the second step (days 6–10), along with other cytokines, including 15 ng/mL rat recombinant granulocyte-macrophage colony-stimulating factor (GM-CSF; STEMCELL), 100 ng/mL SCF, 100 ng/mL FL, 20 ng/mL IL-3 (BioLegend, CA, USA), and 10 ng/mL TPO.

Flow cytometric analysis

The differentiation of HSPCs into megakaryocytic and erythroid lineages was also assessed using the combination of two biomarkers, CD71 and CD41, which are commonly used to define erythroid and MK cells, respectively. Antibodies used in this experiment were anti-CD71-PE antibody (BD Biosciences, San Jose, CA, USA), CD41 polyclonal antibody (Thermo Fisher, MA, USA), and FITC-conjugated goat anti-rabbit IgG secondary antibody (Thermo Fisher). Additionally, erythroid cells were identified and categorized into distinct populations based on HIS49 and CD71 expression as well as cell size as previously described.[27] In brief, 1×10^6 cells were incubated for 30 minutes in 100 μL of PBS containing 0.5% BSA with 0.03 μg of biotin-conjugated anti-rat erythroid antibody HIS49 and 0.03 μg of PE-conjugated anti-rat CD71 (both from BD Biosciences, San Jose, CA, USA). After three washes, the cells were treated with APC-conjugated streptavidin (BD Biosciences). Flow cytometric analyses were conducted using a BD LSR Fortessa flow cytometer (BD Biosciences).

Bioassays and hematological measurements

Hematological parameters were measured using BC2800VET Hematology Analyzer (BC-2800Vet, Mindray Medical International Limited, Shenzhen, China). Serum iron was measured by a chemical colorimetric method with the QuantiChromTM Iron Assay Kit (DIFE-250; BioAssay Systems, CA, USA). Serum transferrin and ferritin concentrations were measured by ELISA kits (ab137993 and ab157732, Abcam, Waltham, MA, USA).

Prussian blue staining

Prussian blue staining was performed to evaluate iron content in the liver and spleen of IDA rats with different treatments. The paraffin-embedded sections were processed through a series of submersions: first in Xylene I for 20 minutes, then in Xylene II for another 20 minutes, followed by Anhydrous ethanol I for 5 minutes, Anhydrous ethanol II for 5 minutes, and finally 75% Ethyl alcohol for an additional 5 minutes. The sections were rinsed three times with tap water and then with distilled water. A Prussian blue staining kit (G1029, Servicebio, Wuhan, China) was used for staining. Equal parts of Prussian blue staining solution A and Prussian blue staining solution B were combined to prepare the staining solution. The slides were immersed in this solution for one hour, followed by two rinses with distilled water. Next, Prussian blue staining solution C was applied to the slides and stained for 3 minutes, then rinsed with running water. The sections were treated with absolute ethanol I for 5 minutes, followed by absolute ethanol II for another 5 minutes, and absolute ethanol III for an additional 5 minutes. Afterward, immerse the sections in Xylene I for 5 minutes, followed by Xylene II for 5 minutes to achieve transparency. Subsequently, the sections were sealed using neutral gum. For image acquisition, NIKON ECLIPSE E100 equipped with the NIKON DS-U3 imaging system (Tokyo, Japan) and the CaseViewer software (3DHISTECH Kft., Budapest, Hungary) were used.

Western blot analysis

Proteins were extracted by incubating the cells in cell lysis buffer (P0013, Beyotime, Shanghai, China) added with protease and phosphatase inhibitors at 1:50 (v/v) (P1045, Beyotime, Shanghai, China). Protein concentration was determined using a BCA protein quantification kit (P0010, Beyotime, Shanghai, China). The proteins were then separated by sodium dodecyl sulfate-polyacrylamide gel electrophoresis. The PVDF membranes were blocked with nonfat milk (5%). Immunoblotting was performed using the primary antibodies phospho-p44/42 MAPK (Erk1/2) rabbit mAb (#4376S, Cell Signaling Technology, MA, USA), p44/42 MAPK (Erk1/2) rabbit mAb (#4695T, Cell

Signaling Technology) and GAPDH (Abcam, Boston, WA, USA). Secondary antibodies used were anti-rabbit IgG, HRP-linked antibody (#7074S, Cell Signaling Technology) and DyLight™ 488 (Thermo Fisher, MA, USA). The phospho-p44/42 MAPK antibodies were stripped from the membrane with Western Blot Antibody Stripping Buffer (Affinibody LifeScience, Wuhan, China) and then the p44/42 MAPK antibodies were incubated. Images were captured using the ChemiDoc™ MP Imaging System (Bio-Rad, Hercules, California, USA) and analyzed using the ImageJ software.

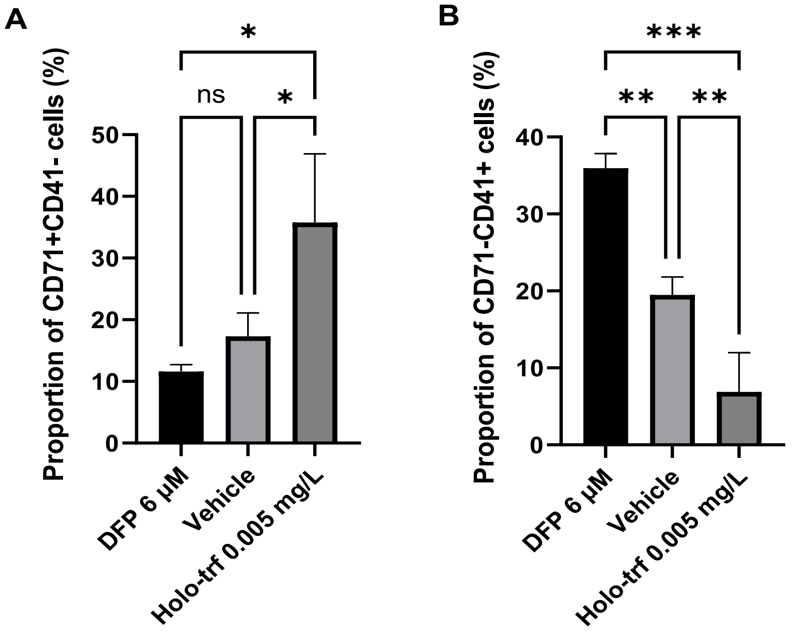

Figure S1. Comparison of the proportion of CD71+CD41- cells (erythroid, A) and CD71-CD41+ cells (MK, B) in HSPCs treated with three different iron concentrations using flow cytometry. Three different iron concentrations were modulated by adding deferiprone (DFP) 6 μM, PBS (Vehicle), or holo-transferrin 0.005 mg/L to the medium. Data are expressed as the mean ± S.D. (n=3 in each group). **P*<0.05, ** *P*<0.01, *** *P* <0.001

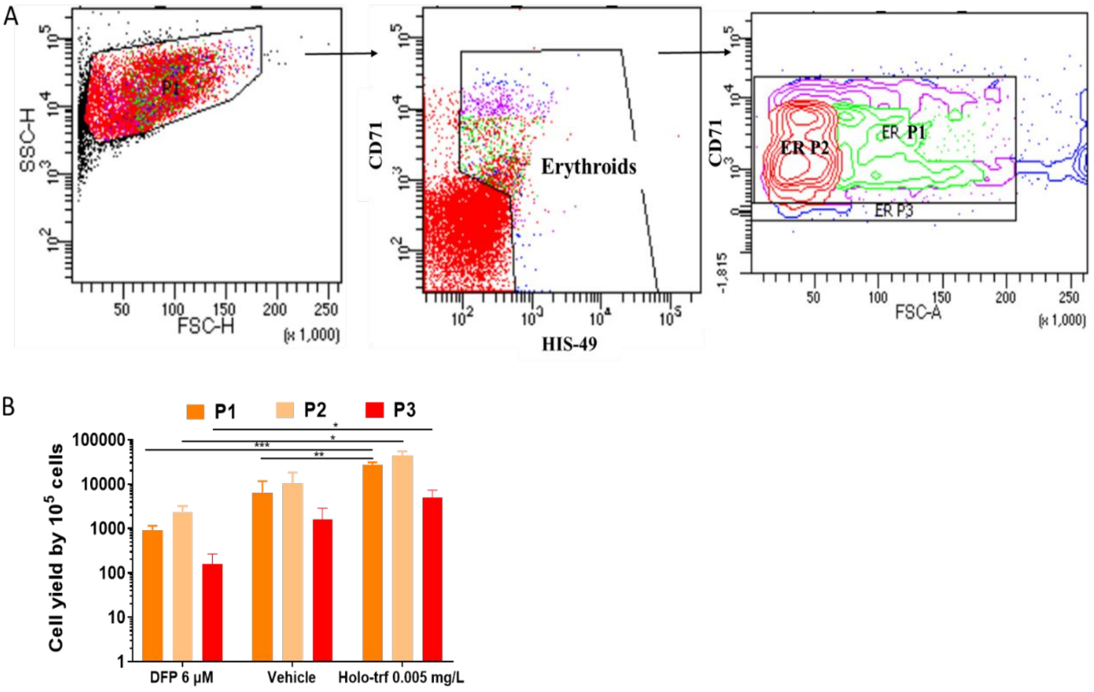

Figure S2. Flow cytometry analysis of erythroid cells. (A) Gating strategy of the flow cytometry to sort and quantify erythroid precursors. EP1, a mixture of proerythroblasts and basophilic, polychromatic and orthochromatic erythroblasts; EP2, orthochromatic erythroblasts; EP3, reticulocytes and mature red blood cells. (B) The changes in the absolute cell numbers of EP1, EP2, and EP3 over time in response to different iron concentrations.

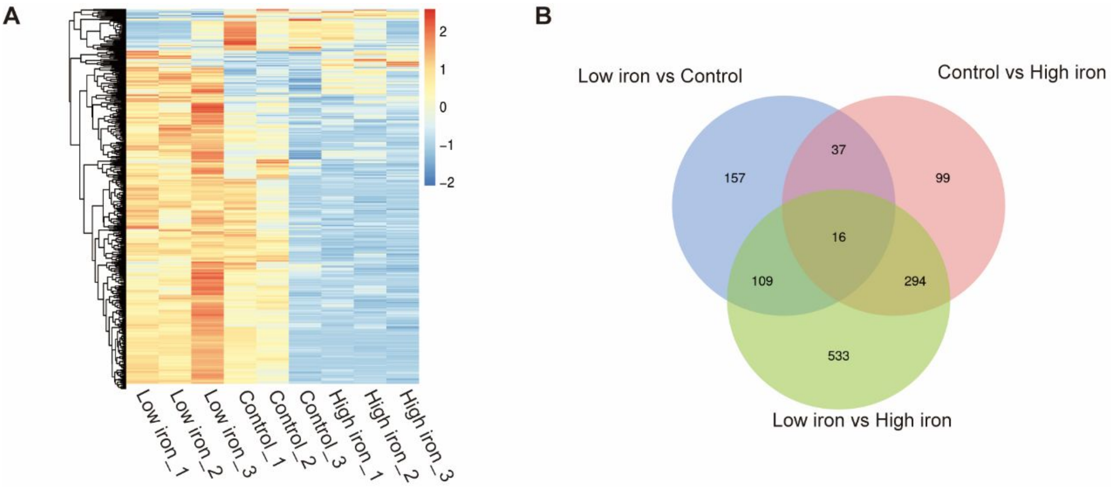

Figure S3. Distinct gene expression profiles in HSPCs treated with three different iron levels. (A) Supervised hierarchical clustering of expression profiles of HSPCs treated with three different iron concentrations. Three different iron concentrations were achieved by adding deferiprone 6 μM (low iron), PBS (control), or holo-transferrin 0.005 mg/L (high iron) to the medium. (B) Venne diagram comparing differently expressed genes between three comparisons of HSPCs cultured at different iron concentrations.

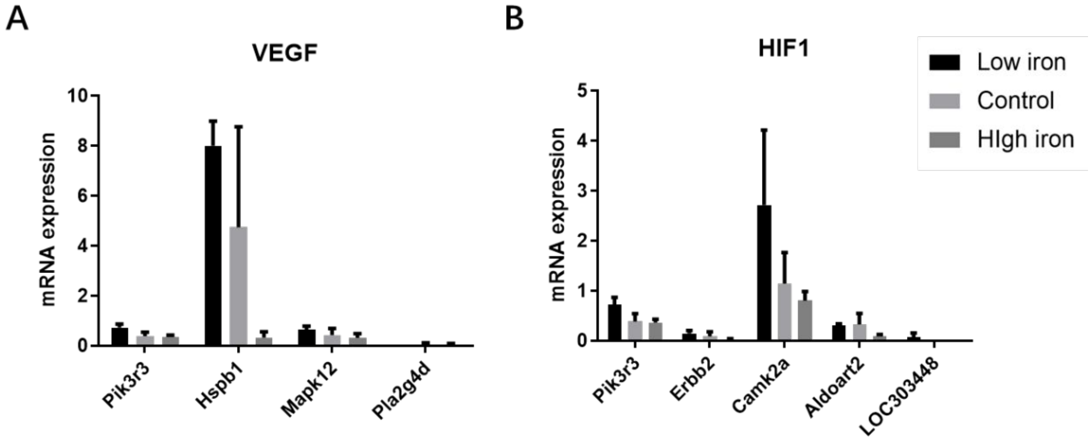

Figure S4. mRNA expression of differently expressed genes related to VEGF(A) and HIF1 (B) in HSPCs cultured at different iron concentrations. Data are expressed as the mean ± S.D. (n=3 in each group).

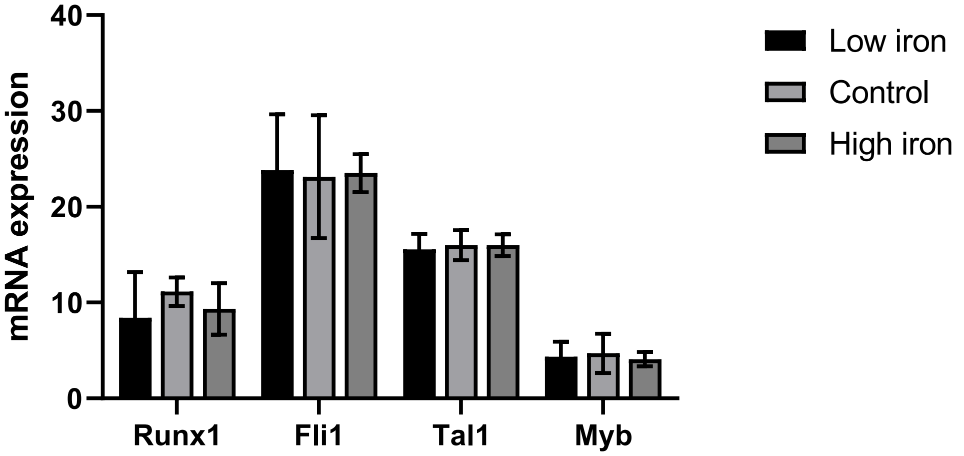

Figure S5. mRNA expression of genes of transcription factors known to regulate megakaryopoiesis and erythropoiesis, including Runx1, Fli1, Tal1, and Myb. Data are expressed as the mean ± S.D. (n=3 in each group).

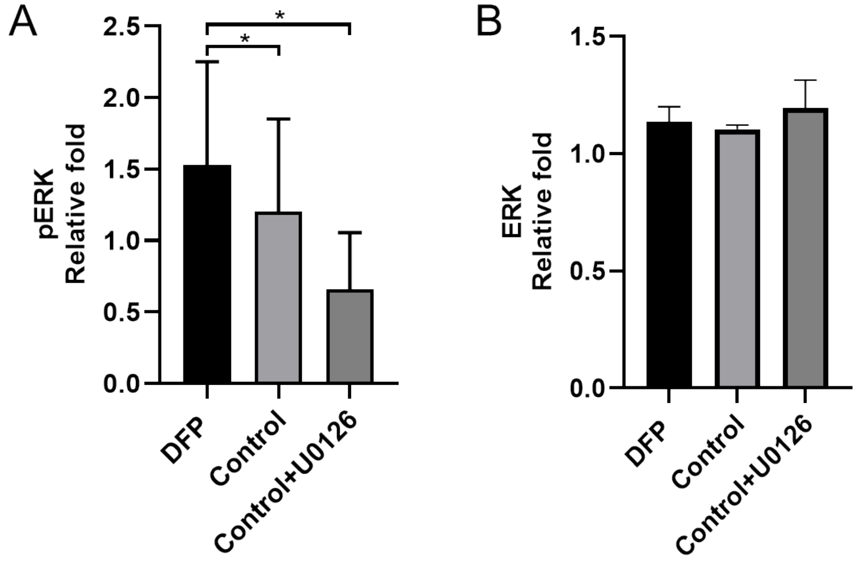

Figure S6. Normalized phospho-ERK (A) and ERK levels (B) in Western blot analysis. Data are shown as mean ± S.D. (A, n=5 in each group; B, n=3 in each group), $*P < 0.05$.

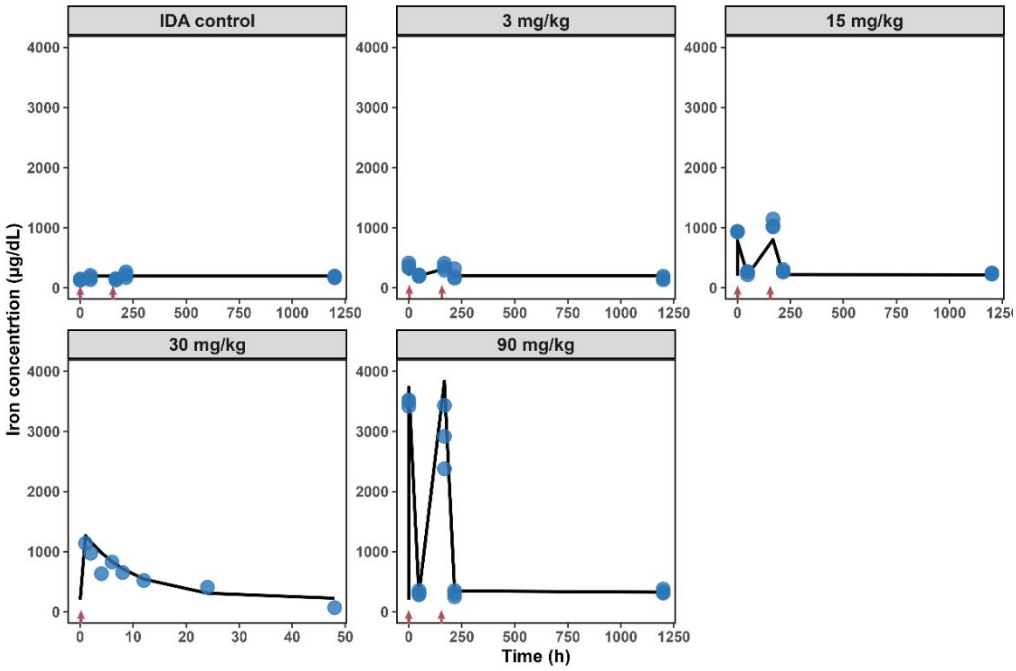

Figure S7. Serum iron concentration versus time profiles in rats. The blue dots represent the observed data, whereas the black lines are model predictions. The red arrows in the line charts represent dosing events.

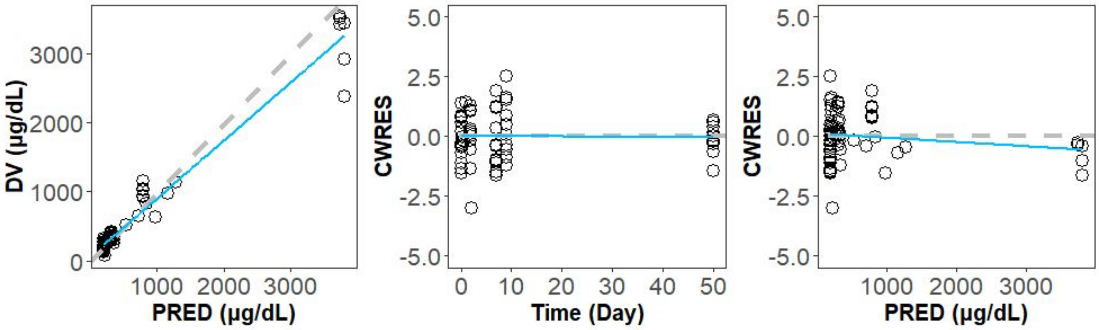

Figure S8. Goodness-of-fit plots of the final PK model. DV, observed values; PRED, model predicted values; CWRES, conditional weighted residuals. The blue lines are the linear regression lines. The gray diagonal (first panels) and horizontal (second and third panels) lines are the y=x and y=0 lines, respectively.

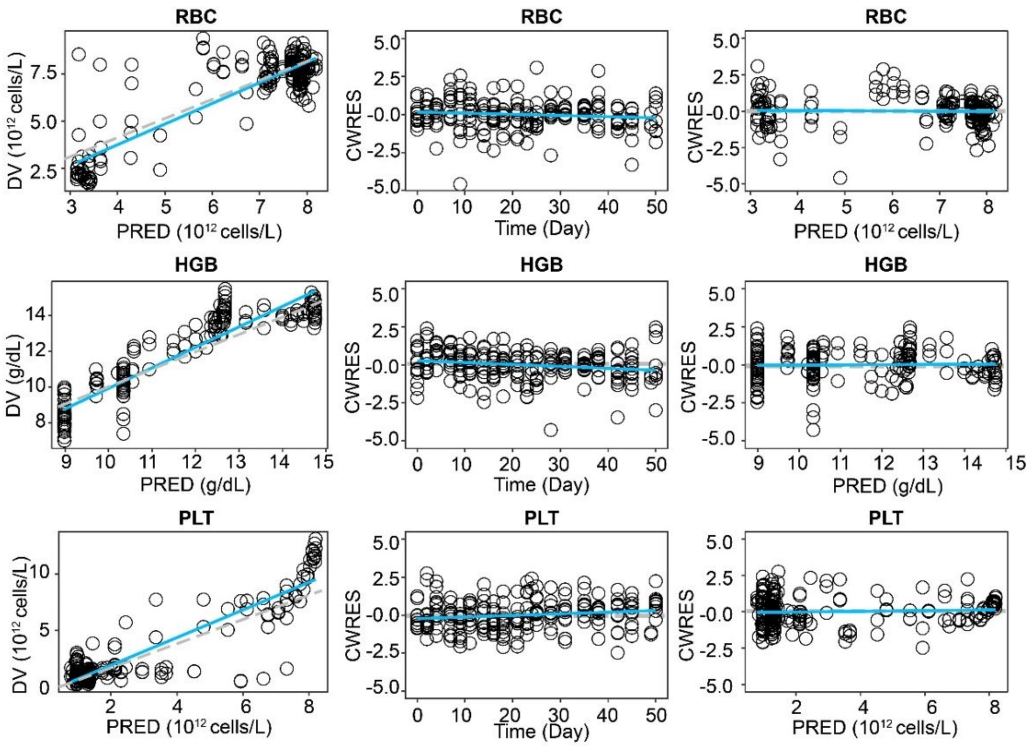

Figure S9. Goodness-of-fit plots of the final PD model. DV, observed values; PRED, model predicted values; CWRES, conditional weighted residuals. The blue lines are the linear regression lines. The gray diagonal (first column) and horizontal (second and third columns) lines are the y=x and y=0 lines, respectively.

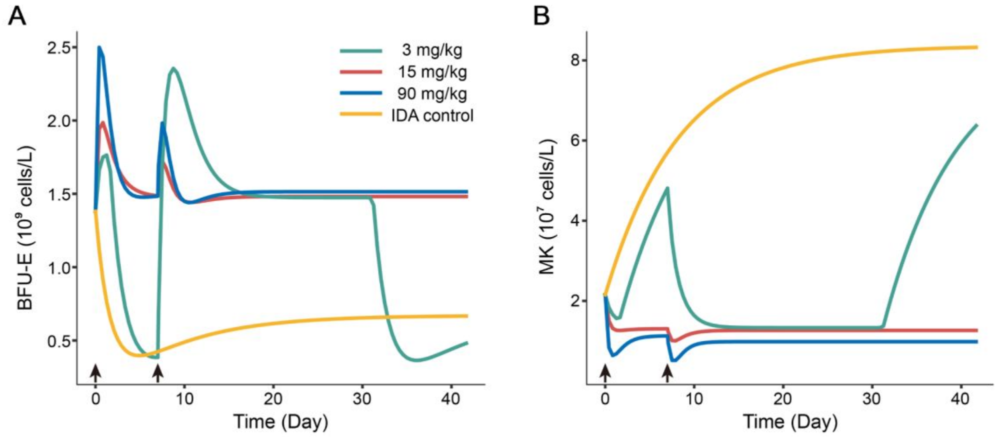

Figure S10. Model-based simulation of the dynamic process of the BFU-E (A) and MK (B) compartment. The black arrows in the line charts represent dosing events.

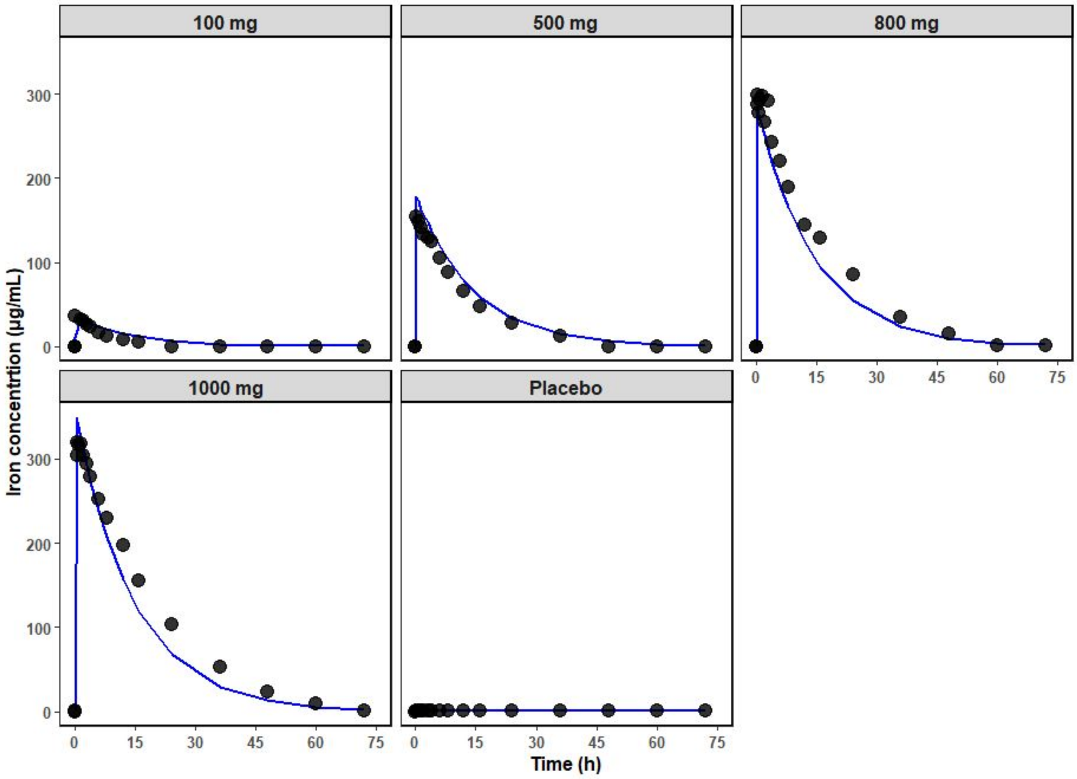

Figure S11. Serum iron concentrations versus time profiles in patients with IDA. The black dots represent observed data in literature.[19] The blue lines represent model predictions.

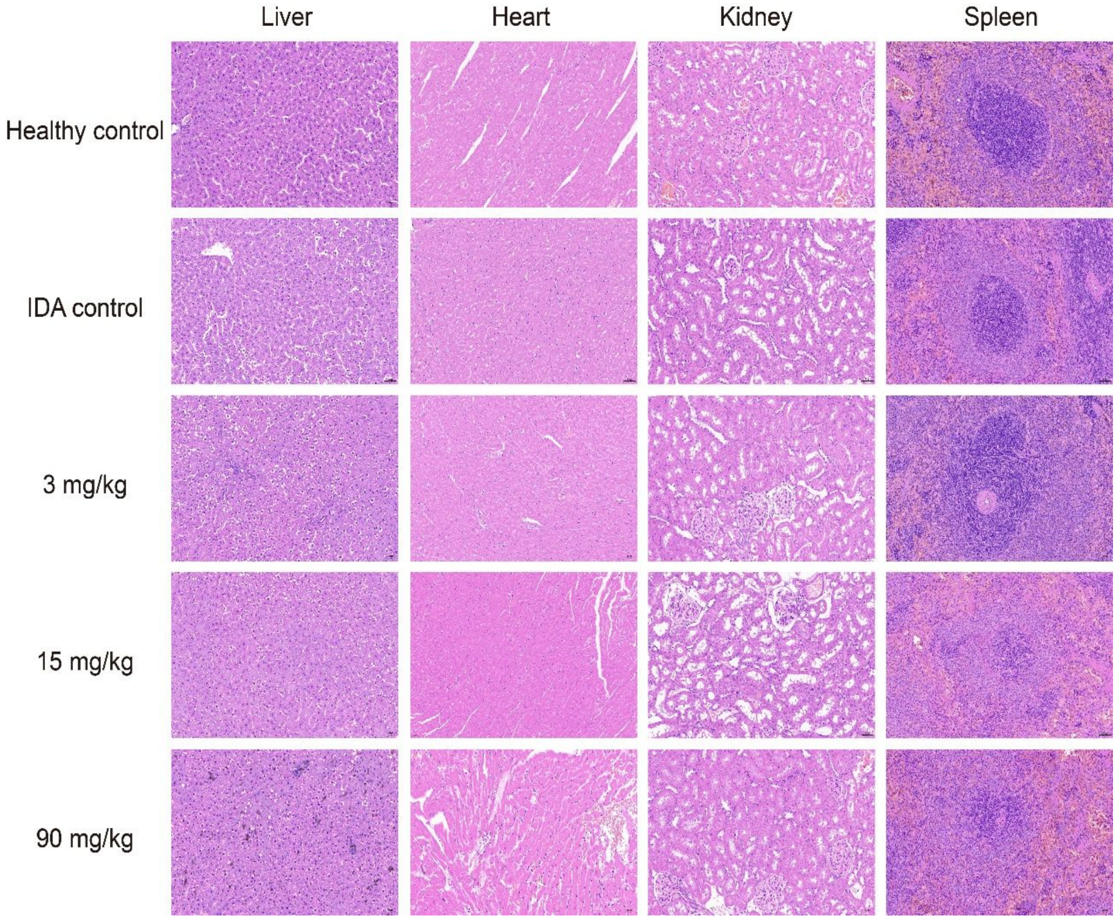

Figure S12. Liver, heart, kidney and spleen sections underwent hematoxylin-eosin (HE) staining. The results shown are from one representative experiment and one representative animal per group. Scale bar, 50 μm.

Table S1. Parameters in humans for the adapted PD model.

Parameters	Definitions	Units	Values (reference)
T_{RET}	Mean residence time for RETs	h	44.8 [21]
T_{RBC}	Mean residence time for RBCs	h	643.2 [22]
RBC_0	Baseline RBCs concentration	$\times 10^{12}$ cells/L	4.4 [21]
T_{MP}	Mean lifespan of megakaryocyte cells	h	216 [23]
T_{PLT}	Mean lifespan of platelets	h	288 [21c]
PLT_0	Baseline platelets in blood	$\times 10^{12}$ cells/L	0.536 [25]
HGB_0	Baseline of HGB	g/L	92.6、86.2 [24]

Table S2. Parameter estimates obtained from the PK model.

Parameter (Units)	Definition	Estimate	RSE (%)
V1 (L/kg)	Volume of distribution of iron in central compartment	2.52	9.5
KIN_{Iron} (mg/h/kg)	Zero-order input rate constant	0.000197	6.7
V2 (L/kg)	Volume of distribution of iron in peripheral compartment	0.687	31.4
Qcp (L/h/kg)	Distribution rate from central to peripheral compartment	0.264	9.4
Qpc (L/h/kg)	Distribution rate from peripheral to central compartment	0.00132	22.4
CL (L/h/kg)	Clearance of iron	0.0001[a]	/
σ_{prop}	Proportional residual error	0.231	8.8

[a]Considering there is no regulated mechanism for the excretion of iron, clearance was fixed to a very small number.

Table S3. Calculation of the secondary parameters based on the baseline condition and PD model estimation.

Parameters	Definition	Calculations	Units
RET0	Baseline RETs concentration	1.41	$\times 10^{12}$ cells/L
NOR0	Baseline NORs concentration	1.41	$\times 10^{12}$ cells/L
CFUE0	Baseline CFU-E concentration	4.41	$\times 10^{10}$ cells/L
BFUE0	Baseline BFU-E concentration	1.38	$\times 10^{9}$ cells/L
HSPCs0	Baseline HSPC concentration	2.08	$\times 10^{9}$ cells/L
KM	First-order rate constant for differentiation of HSPCs into MK	28.7	10^{-4}/h
KIN	Zero-order rate constant for producing HSPCs	405.36	$\times 10^{5}$ cells/(L/h)
MK0	Baseline MK precursor cell concentration	2.16	$\times 10^{5}$ cells/L
